# Prioritizing the sexual and reproductive health and rights of adolescent girls and young women within HIV treatment and care services in emergency settings: a girl-centered agenda

**DOI:** 10.1186/s12978-019-0710-0

**Published:** 2019-05-29

**Authors:** Uchechi Roxo, M. Linda Mobula, Damilola Walker, Allison Ficht, Sarah Yeiser

**Affiliations:** 1 USAID, 2100 Crystal Drive, Arlington, VA USA; 2 USAID, 1300 Pennsylvania Ave NW, Washington, DC USA; 3 UNICEF, 3 UN Plaza, New York City, New York USA

**Keywords:** HIV/AIDS, Adolescent girls and young women, Humanitarian, Conflict, Crisis, Disaster, Framework, Emergency, ART, Sexual and reproductive health, Guidelines, Rights

## Abstract

**Background:**

Extensive documentation exists on a range of negative sexual and reproductive health outcomes and rights violations occurring during humanitarian emergencies. We explore two central questions: Do existing policies, services, and research adequately address the SRH rights, priorities and HIV risks of adolescent girls and young women in emergency settings? What are the missed opportunities for holistically addressing the vulnerabilities experienced by those living with HIV during rapid onset disasters and long term, protracted emergencies? Authors review considerations informing real-time decision making, and highlight missed opportunities to apply a gendered lens in the delivery of AGYW-centered SRHR/HIV services.

**Methods:**

A scoping review identified studies on HIV intervention and outcomes in emergency settings, published in the peer-reviewed literature (2002–2017). This exercise was complemented with a desk review of normative guidance, frameworks, and implementation guidelines on HIV and SRH in emergency responses, and by consultations with subject matter experts.

**Results:**

The existing frameworks and guidance pay scant attention to the sexual reproductive health and rights of young women living with HIV (WLHIV), focusing mainly on prevention of mother to child transmission (PMTCT), antiretroviral therapy (ART), HIV testing services, and linkage to treatment services. Applying a gendered sexual and reproductive health lens to the response offers opportunities to identify critical implementation questions, and highlight promising practices, to better tailor current services for AGYW.

**Conclusions:**

A plurality of competing needs crowds out dedicated time and space to effectively integrate HIV and sexual and reproductive health interventions in emergency settings. Political will is required to advance multi-sectoral cooperation, through joint planning, rights-informed learning and integrative responses, and to promote creative solutions for ART continuation, drug supply and HIV testing, treatment and care. Recent advancements in policy and practice would suggest that a more AGYW-centered response is feasible.

**Electronic supplementary material:**

The online version of this article (10.1186/s12978-019-0710-0) contains supplementary material, which is available to authorized users.

## Background

Humanitarian emergencies have been an episodic disruptor of human immunodeficiency virus (HIV) service delivery, complicating the ability to track patients on antiretroviral treatment (ART), maintain and access care records, rupturing services and supply chains, and even displacing care providers, all of which tend to occur in health systems that may already have known deficits [1]. Further complicating the global HIV response, some affected populations are made more vulnerable to HIV acquisition due to displacement, food insecurity and poverty, which may linger long after periods of crisis end [2].

As explicated in the literature, in a variety of emergency contexts, women and girls are subjected to gross human rights violations stemming from ingrained gender inequality, including reduced access to HIV prevention and reproductive health services, forced occupational exposure, rape as a weapon of war, trafficking, coerced, transactional, and/or survival sex to support self and dependents or family [1, 3–6].

As the frequency and duration of humanitarian emergencies increases, the consequences for women and girls are dire. According to the World Health Organization (WHO), more than half of maternal deaths occur in fragile and humanitarian settings [7]. By UNAIDS estimates, over 1.6 million people living with HIV were affected by humanitarian contexts in 2013, an estimated 60% of whom are women [8]. In the 2017–2018 El Nino-induced drought crisis, an estimated 32 million people were rendered food insecure in the hardest-hit countries of Eastern and Southern Africa, where over 50% of all people living with HIV reside [9, 10].

Recognizing the unique pathways through which humanitarian disasters complicate the global response, a Declaration of Commitment on HIV/AIDS in June 2011 was passed at the United Nations General Assembly Special Session (UNGASS) on HIV/AIDS, stating that, “populations destabilized by armed conflict ... including refugees, internally displaced persons and in particular, women and children, are at increased risk of exposure to HIV infection” [11].

A cursory review of existing manuscripts, frameworks, and guidelines substantiates that adolescent girls and young women (AGYW) with HIV are among the most vulnerable in emergency contexts. Yet, there is little guidance that exists, articulating an integrated and developmentally-sensitive approach to HIV care and treatment, and sexual and reproductive health (SRH). While stakeholders may be able to utilize globally recognized sources such as the U.S. President’s Emergency Plan for AIDS Relief (PEPFAR), the Inter-agency Standing Committee (IASC) Task Force for HIV/AIDS, the SPHERE Network and Joint United Nations Programme on HIV/AIDS (UNAIDS) guidance for service delivery, modifying these for local environments affected by emergencies is largely uncharted territory in countries explored in this review. Even the most robust models of prevention, care and treatment of HIV interventions require simplification and adaptation in a state of crisis or emergency, and revisions or special supplements for countries during complex emergencies, remain as gaps.

The objective of this paper is to examine existing policies and implementation approaches identified through a scoping review in relation to the provision of HIV and Reproductive Health services for AGYW, and to surface recommendations that could transform the current programmatic paradigm. This review also highlights expert suggestions that stakeholders can draw from, to enhance responsiveness to the needs and vulnerabilities in serving this group.

## Methods

### Study selection

Between June 2017 and December 2017, we searched PubMed, Embase, the Cumulative Index of Nursing and Allied Health Literature (CINAHL), and Google Scholar for studies, evaluating whether current emergency response standards and practice adequately respond to the unique risks, vulnerabilities, and developmental needs of adolescent girls and young women (10–24) living with HIV. Key search terms included: HIV and adolescents and humanitarian assistance; Humanitarian medicine; (pediatric HIV) AND (conflict OR humanitarian OR crisis OR emergency); child or youth and HIV and (crisis or conflict); ((adolescent) AND HIV) AND (emergency OR conflict OR crisis OR humanitarian); ((((HIV) AND conflict) OR disaster) OR crisis) OR humanitarian); ((HIV care) AND pregnant) AND (humanitarian OR crisis OR emergency OR conflict); ((HIV care) AND pregnant) AND (humanitarian OR crisis OR emergency OR conflict); famine and HIV.

We assembled a database to document potentially relevant studies. To identify other studies that we might have missed, we reviewed reference lists of articles selected in our primary search.

We included randomized controlled trials (RCTs), cross-sectional, observational cohort, retrospective cohort, pre-post- and qualitative studies. Qualitative studies were included if they were based on primary data collection. Systematic reviews and retrospective studies were screened if they included information on the intersection of SRH across the clinical cascade for AGYW. We also conducted a secondary review of national policies, global frameworks, and international guidelines from countries with a high burden of HIV that experienced significant emergencies in the study period, to augment the prioritization process.

The following inclusion criteria was used to select studies: primary or secondary data on HIV prevention/sexual health, clinical or psychosocial outcomes; reported on a clinical, policy, legal, or programmatic intervention; reported on population outcomes within the context of emergency programming (rapid-onset, slow onset, and/or long-term protracted complex emergencies); or clearly included adolescent girls, young women, or pregnant women aged 10–24 as a study population.

### Data extraction and quality assessment

Abstracts were reviewed by two independent abstractors, who included articles meeting the inclusion criteria stated above. Independent review of the primary study selection was completed by at least one other reviewer. Discrepancies were resolved by consensus. An independent manual search, primarily but not exclusively from manuscript references, identified additional reports for inclusion. Potentially duplicative studies were flagged for review and resolution by group consensus. Full text review by the entire study team and group consensus was required for inclusion of reports on which there was uncertainty.

Data abstraction was conducted by five independent researchers summarizing information about the population, intervention, comparator, outcome, and context/setting into standardized tables. Independent review of the data abstraction was completed by at least one additional researcher.

### Data synthesis and analysis

We examined whether current emergency response standards and practice adequately respond to the unique risks, vulnerabilities, and developmental needs of adolescent girls and young women (10–24) living with HIV.

#### Post-review consultations

We conducted consultations with field-based experts, eliciting expert opinion on program implementation and stakeholder coordination experiences with AGYW programming, HIV and SRH in emergencies, from current or prior work in Democratic Republic of Congo, South Sudan, Côte d’Ivoire, Nigeria, and/or across West and Central Africa. The consultations provided additional perspective, given the paucity of peer reviewed literature on this subject matter, and highlighted areas for further dialogue and inquiry, and areas for future investigation. We aimed to learn the following from these consultations:

1. What are the priority changes deemed most critical for ensuring continuity of HIV treatment and care in emergency settings?
2. What are key barriers to the provision of SRH services for AGYW?
3. What key priorities and opportunities exist to reinforce SRH for HIV positive AGYW and those most at risk?
4. What are some promising or effective approaches, interventions or service delivery models for integrating SRH into HIV programming in a humanitarian settings?
5. What stakeholders, specifically regional and/or international bodies, are best positioned to help advance the HIV-SRH integration agenda to better serve AGYW in conflict-affected areas?

## Results

### Scoping review of HIV service delivery models during emergencies

The search yielded 1250 articles, of which 26 articles met the inclusion criteria for this review. Table 1 provides a summary of the characteristics of these studies, and Table 2 summarizes information about the Population, Concept/Outcome, and Context/ Setting for the 26 studies. We assume that emergency settings include a broad range of settings such as rapid-onset and slow-onset disasters, and complex protracted crises, that do not include conflict. We classified conflict as a specific type of emergency as part of our analysis, observing that it is becoming the most common cause of protracted emergencies, which could potentially impede the delivery or SRH services for PLHIV.

**Table 1:** Studies that met inclusion criteria, characterized by thematic content

	*N = 26*
Population	
Adults (only)	4
Adolescents and children	3
Females (only)	2
General population (all PLHIV)	16
Unspecified	2
Interventions for AGYW Addressed	
Yes	3
No	23
SRHR Addressed	
Yes	9
No	17
Context	
Conflict	13
Post-Conflict	8
Conflict + Post-Conflict	5
Natural Disaster	1
Outcomes	
HIV Prevention	3
HIV Risk	2
HIV Service Utilization	15
ART Retention/Treatment Interruption	5
Reproductive Health	1
HIV-attributable mortality	1

**Table 2:** Study Summary: Population, Context and Outcome Classifications

Study Citation	Population	Design	Context	Outcomes measured	Addresses SRH and AGYW
O’Brien DP, Mills C, Hamel C, Ford N, Pottie K “Universal access: the benefits and challenges in bringing integrated HIV care to isolated and conflict affected populations in the Republic of Congo.” (2009)	PLHIV (adults, adolescents, children)	Retrospective cohort	Conflict	Clinical outcomes of patients on ART	SRH: no AGYW: no
Loko Roka J, Van den Bergh R, Au S, De Plecker E, Zachariah R, Manzi M, Lambert V, Abi-Aad 1, Nanan-N’Zeth K, Nzuya S, Omba B, Shako C, MuishaBaroki D, Basimuoneye JP, Moke DA, Lampaert E, Masangu L, De Weggheleire A. “One size fits all? Standardised provision of care for survivors of sexual violence in conflict and post-conflict areas in the Democratic Republic of Congo.” (2014)	PLHIV (adults, adolescents, children)	Retrospective descriptive cohort	Conflict/post-conflict	High (> 95%) coverage rates of prophylaxes, with follow-up poor; low rates of treatment and/or vaccination completion	SRH: yes AGYW: yes
Atwood KA, Kennedy SB, Shamblen S, Tegli J, Garber S, Fahnbulleh PW, Korvah PM, Kolubah M, Mulbah-Kamara C, Fulton S “Impact of school-based HIV prevention program in post-conflict Liberia.” (2012)	Youth	Feasibility study using randomized control trial	Post-conflict	Knowledge, attitude and practice	SRH: yes AGYW: yes
Holt BY, Effler P, Brady W, Friday J, Belay E, Parker K, Toole M “Planning STI/HIV prevention among refugees and mobile populations: situation assessment of Sudanese refugees.” (2003)	Adults	Qualitative study	Conflict	Knowledge, attitudes and behaviours and HIV/STI prevalence	SRH: yes AGYW: no
Salami, O., A. Buzu, C. Nzeme “High Level of Adherence to HAART Among Refugees and Internally Displaced Persons on HAART in Western Equatorial. Region of Southern Sudan” (2010)	PLHIV (adults, adolescents, children)	Cross-sectional study	Conflict	ART Adherence	SRH: no AGYW: no
Patel S, Schechter MT, Sewankambo NK, Atim S, Oboya C, Kiwanuka N, Spittal PM “Comparison of HIV-related vulnerabilities between former child soldiers and children never abducted by the LRA in Northern Uganda” (2013)	Children	Cross-sectional study	Post-conflict	HIV Prevalence	SRH: yes AGYW: no
Wilhelm-Solomon M “Challenges for antiretroviral provision in northern Uganda” (2010)	PLHIV (adults, adolescents, children)	Commentary	Conflict, post-conflict	HIV service utilization	SRH: no AGYW: no
Yoder RB, Nyandiko WM, Vreeman RC, Ayaya SO, Gisore PO, Braitstein P, Wiehe SE. “Long-term impact of the Kenya post-election crisis on clinic attendance and medication adherence for HIV-infected children in western Kenya” (2012)	Children < 14	Retrospective cohort	Conflict, post-conflict	ART Adherence	SRH: no AGYW: yes
Goodrich S, Ndege S, Kimaiyo S, Some H, Wachira J, Braitstein P, Sidle JE, Sitienei J, Owino R, Chesoli C, Gichunge C, Komen F, Ojwang C, Sang E, Siika A, Wools-Kaloustian K “Delivery of HIV care during the 2007 post-election crisis in Kenya: a case study analyzing the response of the Academic Model Providing Access to Healthcare (AMPATH) program” (2013)	PLHIV (adults, adolescents, children)	Case study	Post-conflict	HIV service utilization	SRH: no AGYW: no
Bamrah S, Mbithi A, Mermin JH, Boo T,Bunnell RE, Sharif S, Cookson ST “The impact of post-election violence on HIV and other clinical services and on mental health-Kenya, 2008” (2012)	Adults	Descriptive study	Conflict	ART Adherence	SRH: no AGYW: no
Pyne-Mercier LD, John-Stewart GC, Richardson BA, Kagondu NL, Thiga J, Noshy H, Kist N, Chung MH “The consequences of post-election violence on antiretroviral HIV therapy in Kenya. AIDS Care” (2011)	Adults	Mixed methods retrospective review	Post-conflict	ART Adherence	SRH: no AGYW: no
Unge C, Södergård B, Thorson A, Ragnarsson A, Carter J, Ilako F, Waweru M, Ekström AM. “HIV treatment in times of civil strife: serious threats to antiretroviral drug access in the Kibera slum following the Kenyan elections” (2008)	Unspecified ages	Qualitative study	Post-conflict	HIV service utilization	SRH: no AGYW: no
Reid T, van Engelgem I, Telfer B, Manzi M “Lessons learned (programmatic) from MSF’s three primary health care centers, including HIV treatment and support services in Kibera slum” (2008)	Unspecified ages	Commentary	Post-conflict	HIV service utilization	SRH: no AGYW: no
Anthonj C, Nkongolo OT, Schmitz P, Hango JN, Kistemann T “The impact of flooding on people living with HIV: a case study from the Ohangwena Region, Namibia” (2015)	PLHIV (adults, adolescents, children)	Qualitative study	Natural disaster	HIV service utilization	SRH: no AGYW: no
Noden BH, Pearson RJ, Gomes A “Age-specific mortality patterns in Central Mozambique during and after the end of the Civil War” (2011)	PLHIV (adults, adolescents, children)	Retrospective cohort	Conflict	Mortality	SRH: no AGYW: no
Mendelsohn JB, Schilperoord M, Spiegel P, Ross DA “Adherence to antiretroviral therapy and treatment outcomes among conflict-affected and forcibly displaced populations: a systematic review” (2012)	PLHIV (adults, adolescents, children)	Systematic review	Conflict/post-conflict	ART Adherence	SRH: no AGYW: no
Culbert H, Tu D, O’Brien DP, Ellman T, Mills C, Ford N, Amisi T, Chan K, Venis S “HIV treatment in a conflict setting: outcomes and experiences from Bukavu, Democratic Republic of the Congo” (2007)	PLHIV (adults, adolescents, children)	Descriptive study	Conflict	HIV service utilization	SRH: no AGYW: no
Simon, S “Review of International Federation of Red Cross and Red Crescent Societies (IFRC) material on HIV and AIDS and sudden onset emergencies” (2008)	PLHIV (adults, adolescents, children)	Program report	Natural disaster	HIV service utilization	SRH: no AGYW: no
Da Waal A, Klot J, Mahajan M “HIV/AIDS, Security and Conflict: New Realities, New Responses” (2009)	PLHIV (adults, adolescents, children)	Commentary	Post-conflict	HIV service utilization	SRH: yes AGYW: no
Spiegel, P “HIV in emergencies – much achieved, much to do” (2010)	PLHIV (adults, adolescents, children)	Commentary	Conflict/post-conflict	HIV service utilization	SRH: yes AGYW: no
Spiegel, P “The effects of antiretroviral therapy on HIV prevalence in conflict situations: not yet there.” (2009)	PLHIV (adults, adolescents, children)	Commentary	Conflict	HIV service utilization	SRH: no AGYW: no
Spiegel, P “Populations: Dispelling myths and taking action.” (2004)	PLHIV (adults, adolescents, children)	Commentary	Conflict	HIV service utilization	SRH: yes AGYW: no
Whitmill J, Blanton C, Doraiswamy S, Cornier N, Schilperood M, Spiegel P, Tomczyk B “Retrospective analysis of reproductive health indicators in the United Nations High Commissioner for Refugees post-emergency camps 2007–2013” (2016)	Women	Retrospective analysis	Conflict	Reproductive health indicators	SRH: yes AGYW: no
Griffiths K, Ford N “Provision of antiretroviral care to displaced populations in humanitarian settings: a systematic review.” (2013)	PLHIV (adults, adolescents, children)	Systematic review	Conflict, natural disaster	HIV service utilization	SRH: no AGYW: no
Hankins C A “Transmission and prevention of HIV and sexually transmitted infections in war settings: Implications for current and future armed conflicts” (2002)	PLHIV (adults, adolescents, children)	Commentary	Conflict	MISP, family planning indicators and HIV Prevention, interventions for injecting drug use	SRH: yes AGYW: no
Ssonko C, Gonzalez L, Mesic A, Silveira de Fonesca M, Achar J, Safar N, Martin B, Wong S, Casas E “Delivering HIV care in challenging operating environments: the MSF experience towards differentiated models of care for settings with multiple basic health care needs” (2017)	PLHIV (adults, adolescents, children)	Descriptive analysis	Conflict	HIV service utilization	SRH: no AGYW: no

There was a paucity of evidence on the intersection of sexual and reproductive health and HIV services for AGYW in emergency contexts. No article assessed specific interventions - instead we saw a combination of review articles, case studies and commentary pieces.

Given the heightened risk of sexual violence, sexually transmitted infections (STI), termination of pregnancy and early loss, premature delivery, stillbirths, delivery-related complications, and neonatal and maternal mortality, there is a missed opportunity for cross-examination of these issues in emergency settings beset by high HIV prevalence or low coverage with HIV and/or SRHR interventions.

### Secondary review of existing policy, normative guidelines, and global standards

The content of key global frameworks, normative guidelines and global standards for emergency- and/or disaster-related action, response and recovery, was reviewed for clarity on existing guidance on programmatic decision making in emergency/post-emergency and resource constrained environments. Table 3 summarizes the findings.

**Table 3:** Key Global Frameworks

Year	Author or Organization	Framework Title
2017	World Health Organization (WHO)	A Strategic Framework for Emergency Preparedness [12]
2017	United Nations Office for Disaster Risk Reduction (UNISDR)	Build Back Better in Recovery, Rehabilitation and Reconstruction [13]
2017	Lo, S.T.T. et al.	Health Emergency and Disaster Risk Management: Developing the Research Field within the Sendai Framework Paradigm [14]
2016	UN Office for the Coordination of Humanitarian Affairs (OCHA), International Organization on Migration (IOM)	The Grand Bargain - A Shared Commitment to Better Serve People in Need [15]
2016	World Health Organization (WHO)	Bangkok Principles for the implementation of the health aspects [16]
2013 & 2016	Federal Emergency Management Agency (FEMA)	National Disaster Recovery Framework (& 2nd version) [17]
2015	Inter-Agency Standing Committee (IASC)	Reference Module for Cluster Coordination at Country Level [18]
2015	Global Facility for Disaster Reduction and Recovery (GFDRR), World Bank, United National Development Program (UNDP)	Guide to Developing Disaster Friendly Frameworks [19]
2015	UNISDR	The Sendai Framework for Disaster Risk Reduction (2015–2030) [20]
2014	UNISDR	Post 2015 Framework for Disaster Risk Reduction [21]
2014	Overseas Development Institute (ODI)	Disaster Resilience for Sustainable Development [22]
2013	IASC	IASC Policy on Protection in Humanitarian Action [23]
2012	IASC	IASC Task Team on Accountability to Affected Populations and Protection from Sexual Exploitation and Abuse (AAP/PSEA) [24]
2011	IASC	IASC Transformative Agenda [25]
2011	FEMA	National Response Framework [26]
2006	UNHCR & UNICEF	HIV/AIDS, Conflict and Displacement Conference Report [27]
2005	UNISDR	The Hyogo Framework for Action 2005–2015: Building the Resilience of Nations and Communities for Disasters [28]
1999	UNISDR	The International Strategy for Disaster Reduction: A Safer World in the twenty-first Century: Disaster and Risk Reduction [29]
1994	UNISDR	The Yokohama Strategy for a Safer World [30]
1989	UNISDR	The International Framework of Action for the International Decade for Natural Disaster Reduction (IDNDR) [31]

We identified 20 global frameworks that address emergency response and recovery. These frameworks generally focused on planning, activation, coordination, monitoring, governance and information sharing, with attention paid to the need to enhance programming directed at the nexus between the humanitarian and development sectors. Considerations on programming responses to hunger, poverty, education, water, shelter, and ecosystem management were consistently addressed. Authors ascertained that the normative guidance is generally weak on sexual and reproductive health, HIV and the specific developmental needs of adolescent girls and young women. Among the frameworks listed, none mentioned HIV explicitly or gave specific recommendations, although a few (five of the 20 identified) did mention health, healthcare, or the health system peripherally.

We reviewed established normative sources around which public health emergency response practitioners organize their health and HIV/AIDS efforts. These include: the Inter-agency Standing Committee Guidelines for HIV AIDS Interventions in Emergency Settings [32]; the Sphere Minimum Standards Standards in Humanitarian Response [33], the Minimum Initial Service Package for Reproductive Health of the InterAgency Working Group on Reproductive Health in Crises [34]. In addition to these, guidance on related issues is included in: the Consensus statement on delivering antiretroviral drugs in emergencies: neglected but feasible [35]; the Inter-agency Standing Committee Guidelines on Mental Health and Psychosocial Support in Emergency Settings [36]; the Guidelines for Gender-based Violence Interventions in Humanitarian Settings of the IASC Reference Group on Gender and Humanitarian Action [37]; and the IASC 2017 Gender Handbook for Humanitarian Action of the IASC Reference Group on Gender and Humanitarian Action [38].

The Minimum Initial Service Package for Reproductive Health (MISP) is a compilation of life saving measures, designed to prevent and manage the consequences of sexual violence; prevent excess maternal and newborn morbidity and mortality; reduce HIV transmission; and plan for comprehensive RH services beginning in the early days and weeks of an emergency [34]. As part of MISP, it is essential to train prior to a disaster in order to be ready to deploy at the regional, state, and district level. Training should center on interventions for SRH, GBV, HIV, and STIs [34]. In the context of an emergency, MISP should be prioritized by the Health sector/ cluster and the Ministry of Health (MoH). The role played by the health sector/cluster in implementing the MISP is outlined in the IASC Health Cluster tools and guidance [39, 40].

Vigilant attention to prevention needs in the context of emergency settings could provide the additional benefit of detecting of acute HIV infection, a need which is all the more critical for AGYW in the reproductive age group. Existing frameworks identify (sometimes indirectly) the specialized need for the availability of post-exposure prophylaxis (PEP), and more recently, for pre-exposure prophylaxis (PrEP). The WHO’s “Clinical Management of Rape (CMR) Survivors: Developing Protocols for use with refugees and internally displaced persons - Revised Edition” provides specific protocols and guidance on the use of PEP, as well as treatment of sexually transmitted diseases, without specifically mentioning methods to reach AGYW. CMR is an essential component of MISP and should be included as part of integrated Health and GBV programming. The guidance stipulates conditions under which health providers should assess high HIV risk, based on general HIV prevalence and known or unknown perpetrator risk [41]. This guidance should be adapted and scaled up to reach AGYW through medical and psychological services.

A 2014 study (Roka et al.) on patterns of sexual violence, survivor characteristics, and components of the Médecins Sans Frontières (MSF) response across two provinces (one conflict, one post conflict) in the Democratic Republic of Congo found that only 46% of survivors (*n* = 671) reached the clinic in the critical 72 h window. Primary reasons for the delay in seeking services were fear, shame, and lack of knowledge on the available treatments/services. Critically, follow up for prevention services (HIV testing after postponing initial test and completion of PEP), however, was low [42]. Psychological counseling, though provided at intake and follow up visits, was underutilized due to the overarching challenges of retaining survivors. Appropriate messaging and education through community and NGO outreach can help quell these fears and increase knowledge on available interventions, empowering survivors to reach crucial services.

MSF, has been a leader in documenting broad programmatic experiences, and in consultative engagement with HIV implementers in select countries. MSF’s contributions are noteworthy because they offer a comprehensive package of services to sexual violence survivors with high uptake in some settings [42], comprised of:

...A full medical examination including a genital and/or anal examination, opt-out offer of HIV counselling and testing, and pregnancy testing), medical care (emergency contraception (for all females aged 12–45, presenting within 120 hours after rape), prophylaxis for STIs (STI – for all rape survivors), HIV PEP (for all rape survivors presenting within 72 hours), vaccination for hepatitis B and tetanus, and wound care if indicated), psychological counselling, preparation of a medico-legal certificate, medico-legal support if requested, and safe shelter and external referral for social assistance for specific cases... [42].

The Interagency Task Team HIV in Humanitarian Emergencies [43] has recommended at a minimum, assuring a continued ARV supply for pregnant and breastfeeding women known to be HIV positive and on ARVs, and access to safe and clean deliveries, infant feeding counselling and perinatal prophylaxis for HIV exposed infants.

The IASC Task Team on Accountability to Affected Populations (AAP) and Protection from Sexual Exploitation and Abuse (PSEA) was established in 2012 in order to foster a culture of accountability and protection from sexual exploitation and abuse at all levels of the humanitarian system [24]. It encourages institutionalization of AAP and PSEA within humanitarian organizations, and supports operationalization of AAP and PSEA at the collective level as well as at the level of individual agency, critical elements of responsive programmes to which humanitarian actors have been recently awakened.

## Discussion

While HIV is often viewed as a lesser priority in emergency contexts - particularly due to other competing demands, the weakened health infrastructure and low availability of medical professionals - skilled cadres who focus on HIV treatment and care needs of PLHIV, can increase clinical capacity and help alleviate workforce constraints. Supplementing the health cluster and disaster response teams with HIV specialists can be beneficial beyond the scope of HIV and AGYW services. A 2002 study by O’Brien et al. in the Democratic Republic of Congo found that “doctors caring for HIV patients also worked in the adult medical, paediatric, emergency and TB wards, and counselors undertook general psychosocial counselling for HIV negative people (e.g. post-traumatic counselling) as well as HIV related counselling and education activities [44].” Additional doctors, counselors, and laboratory technicians can be included as part of the staff portfolio and assist with training of trainers (TOT), attending to routine medical needs as well as the physical and mental health needs of AGYW.

As HIV services are built out for AGYW and particularly survivors of sexual violence in emergency contexts, follow-up must be stressed as a vital aspect of programming. This support can also take the form of community-led outreach. In an example from South Sudan, a female-centered WASH activity (originally designed to assist girls at-risk) increased their vulnerability, when sexual harassment and assaults were common and grossly concealed at target sites. After women and girls self-reported sexual assault during trips to the latrines and water collection points in the evenings, they organized themselves for group water collection and recurring meetings, forming a support system for those victimized and others. This local forum enabled the implementing partners to offer medical, psychosocial and case management services [45]. Most importantly, a spillover effect of AGYW meeting together, this intervention encouraged a subset to proactively seek services.

The imperative to reach AGYW must be rooted in rapid assessment of specific needs, meaningful engagement of those living with HIV and affected post-conflict, and where appropriate integrated services. Identification and segmentation of a subpopulation of young women in each West and Central African country looks different and is critical to determine, develop and deliver the right (and most) impactful interventions. For example, although country dependent, understanding the profiles of AGYW and young mothers is a critical step before reaching consensus about the most essential interventions. Varying contexts will call for tailoring of HIV and SRH service delivery strategies to adolescents within the general population, those living with HIV, first time mothers, AGYW engaged in sex work, and other competing priorities prone to require support.

As stressed in the MISP and IASC frameworks, coordination and continuous communication among partners working in HIV and SRH are both paramount to support host country efforts. Host governments, US Government/PEPFAR, the Global Fund to Fight AIDS, Tuberculosis, and Malaria field teams, UNAIDS coordinator, a member of the humanitarian country team, the Health Cluster, World Food Program, and lastly a host of donor-funded implementers and humanitarian actors, need to exchange information. Having one or multiple agencies serve in a convenor role may help facilitate timely and accurate reporting of current and shifts in activities. This enables all to have a picture of geographic, intervention and resource areas. In addition to these stakeholder meetings, feedback from one regional West and Central Africa health office Director, also underscored the need to adapt technical assistance, monitoring and supervisory approaches and simplify these based on what is the most feasible in one country situation.

The authors mapped a number of issues below based on their predominance across study content. For the subject matter mentioned as requiring special attention, only four (4) articles covered programmatic considerations in relation to AGYW, while promising and evidence-informed interventions were largely lacking for targeting this group. The majority of studies, focusing on HIV service provision, address approaches to reach the general population and called for alternative programmatic solutions when these are disrupted. Therefore, a key shortcoming of the studies is the number of unanswered questions -- what is being done to reach young girls and what can be learned about how AGYW living with HIV overcome service delivery barriers; and for those that ably seek, receive and remain linked to treatment, care and SRH services in countries plagued by insecurity, what factors are at play?

Interventions tailored for AGYW with known HIV status and those at high risk should take into account survivors of rape, sexual violence, exploited young girls and women engaged in sex work and transactional sex. Specifically, increased research and documentation is needed on how to reach young adolescents through facility- or community based care, through their social and sexual networks, and through task-shifting support. The importance of integrating SRH and HIV services in West and Central Africa has been highlighted in high level forums such as the 2018 international AIDS conference alongside the threats to AGYW health and approaches to advance the HIV prevention and child protection agenda. While the number of Calls to Action [46] for addressing risks, vulnerabilities and developmentally-appropriate services for HIV positive AGYW living in conflict prone countries are increasing, there is limited documentation of how humanitarian responses have applied a AGYW-friendly and gender lens to HIV services systematically and routinely in Cote d’Ivoire, Haiti, Nigeria, Democratic Republic of Congo, and South Sudan -- five environments facing continuous periods of fragility with implications for the health sector response (see Additional file 1).

Given the paucity of peer-reviewed literature on this subject matter, we aimed to ground our scoping review with the perspectives of practitioners who have substantial experience coordinating HIV and SRH services in emergency settings. In February of 2017, we held individual phone interviews with seven experts and one in-person meeting with key global health institutions, including: UNAIDS, UNICEF, USAID West and Central Africa. We aimed to learn: key priorities to reinforce SRH messages and services within HIV programs for AGYW and core recommendations for carrying these out, based on their collective implementation experience.

All respondents ranked obstetric and antenatal care for pregnant women living with HIV; antiretroviral drugs and other HIV commodities security and GBV prevention and services for rape services, as critical areas of focus. Supporting AGYW through household and community mobilization to address their heightened risk for of HIV from rape and sexual exploitation was re-emphasized repeatedly as was the partner coordination and host government leadership intangibles.

Five common themes for prioritization within emergency settings and programs tailored to young AGYW and those of reproductive age, were identified by experts:

• Women’s and Girls’ Protection and Violence Response, including questions around effective responses and realities of some operational constraints/limitations of targeting adolescent girls and other survivors of rape, sexual and intimate partner violence (as well as controlling for unintended and adverse outcomes from cash transfers, safe spaces and other programs)

• Pregnancy and PMTCT, with a focus on the increased vulnerability of adolescents, and approaches for ensuring service providers can cater to needs of mother and child with known and unknown HIV status

• Poor access to the health system, highlighting the need for more research on access to contraceptives, ART drugs and viral load monitoring through service delivery platforms in an emergency setting while addressing compounded stigma, discrimination and varying knowledge, attitudes and practices of AGYW

• Mental health and psychosocial support, particularly a need for more evidence on the most feasible, cost effective and useful individual as compared to peer-group based interventions.

• Voice, accountability, and empowerment, with a concern for new modalities to amplify and leverage these attributes so they can translate into gains across community mobilization, quality of care, and agency of AGYW while mobile, displaced and/or resettled.

The content of our consultations provided practical insights on ways to align with the key tenets outlined in the 2015 Interagency Task Team on HIV in Humanitarian Emergencies guidance [43]. Their recommendations underscore the need for integrating SRH and rights into HIV services for AGYW, but also mandate a multi-stakeholder response to:

• Identify appropriate platforms (provider-based, individualized/peer and/or community-initiated) for AGYW to understand the range of (or the most critical) services and help facilitate destigmatized care and treatment support. For AGYW affected by violence, improve integration of GBV responses with Health programming

• Prioritize ART and life saving medicines for those living with HIV and pregnant to prevent mother to child transmission and strengthening cross border programming and mobile service outlets that aim to reach AGYW

• Strengthen the emphasis on PMTCT for pregnant women within the MISP, particularly access to modern contraceptive options for women living with HIV, which continues to be a key underemphasized element of effective PMTCT programming. HIV development actors should ensure continued access to PMTCT services by rapidly reprogramming their activities during emergencies, while ensuring that critical elements of the MISP are being provided for women in need of RH services;

• Share culturally-sensitive, age- and language- appropriate information with girls and their caregivers, including ways to prevent, treat and manage HIV and STIs, space pregnancies, address menstrual hygiene management (MHM), negotiate safer sex, and on how to access services, part of holistic programming

• Better understand and respond to the varied nuances of programming for hidden AGYW populations, including female combatants, sex workers, girls engaged in transactional sex, girls in forced and/or early marriages, women with substance use dependencies, transgender women, and other special sub groups, rather than assuming homogeneity across females seeking or receiving HIV services

• Support mental health services as part of outreach and in clinic settings, including creative health workforce solutions that address mental health needs. Equip them, and other providers (plus lay workers) through training and task shifting strategies, thus expanding the pool of human resources for health in facilities and communities

• Ensure humanitarian actors, HIV and other health implementing partners remain in consistent communication and when needed engage in joint planning through the Health Cluster and other platforms

• Explore novel data solutions and architecture at all levels, decentralized to national, to improve tracking across settings and safeguard patient safety, including mobile data, biometrics, and/or cloud-based health information systems

• Update global guidelines and policies to reflect strategies and implement country-specific operational plans for integrated HIV and SRH services in the humanitarian emergency setting

• Maintain flexibility with funding sources and openness to redirection, reallocation and resources and where possible integrating SRH and HIV in other routine programs deemed as first tier priority in a given country (e.g., nutrition, water, sanitation and health)

## Conclusions

Opportunities abound to advance a prioritization agenda for AGYW-centered care at policy, program and strategic information levels to improve quality and scope of services, especially in emergency settings. The authors assert that applying a SRH lens in even the most challenging operating environments, is as essential as ensuring a well-coordinated, locally led multi-sectoral response, if this special population is not to be left behind. Clearly defining in practical terms approaches that prioritize identifying (then linking) adolescent girls and young mothers living with HIV and at most risk to services, remains a critical step. Integrating humanitarian partner activities with the HIV response, may offer the best platform for reaching AGYW in countries affected, if client-centered. In order to go beyond topical gaps, identified from the scoping review, country specific plans, investments and research cannot be confined to a reliance on Calls to Action and recent guidelines alone. Learnings from what is working in SRH services in complex emergency settings in Africa, must be captured by implementers and nuanced at meeting the needs of AGYW of different ages. Rigorous documentation of effective practice requires more attention, particularly in rapid/sudden onset emergencies. Stakeholders must maintain a heightened awareness of sociological context of a particular emergency setting while adapting interventions for targeting AGYW to access services.

A French translation of this article has been included as [see Additional file 2].

A Portuguese translation of the abstract has been included as [see Additional file 3].

## Additional files

Additional file 1: Country Snapshots of HIV Service Gaps and Opportunities in Emergency Setting. (PDF 58 kb)

Additional file 2: Translation of this article into French. (PDF 312 kb)

Additional file 3: Translation of the abstract of this article into Portuguese. (PDF 97 kb)

## Abbreviations

AAP: Accountability to Affected Populations
AGYW: Adolescent girls and young women
ART: Antiretroviral therapy
GBV: Gender-based violence
HIV: Human immunodeficiency virus
IASC: Inter-agency Standing Committee
MHHM: Menstrual Health Hygiene Management
MISP: Minimum Initial Service Package for Reproductive Health
MoH: Ministry of Health
MSF: Medecins Sans Frontieres
PEP: Post-exposure prophylaxis
PEPFAR: Resident’s Emergency Plan for HIV/AIDS Relief
PMTCT: Prevention of mother to child transmission
PrEP: Pre-exposure prophylaxis
PSE: Protections from sexual exploitation and abuse
RCT: Randomized control trial
RH: Reproductive Health
SRH: Sexual and Reproductive Health
TB: Tuberculosis
TOT: Training of trainers
UNAIDS: Joint United Nations Programme on HIV/AIDS
UNGASS: United Nations General Assembly Special Session
USAID: United States Agency for International Development
WHO: World Health Organization

## Authors’ information

UR is a Regional Advisor in the Office of HIV/AIDS, Global Health Bureau, US Agency for International Development (USAID). LM is a Public Health and Infectious Disease Advisor in the Division of Preparedness, Strategic Planning and Mitigation of the Office of U.S. Foreign Disaster Assistance (OFDA) at USAID. DW is Sr. Advisor for Adolescent HIV in the HIV and AIDS Section, Programme Division, UNICEF New York. AF is the Pediatric and Maternal Clinical Branch Program analyst for USAID in the Global Health Bureau, Office of HIV/AIDS and SY is the Integrated Services Program Analyst for USAID in the Global Health Bureau, Office of HIV/AIDS.

## References

[1] Spiegel P (2004). HIV/AIDS among conflict-affected and displaced populations: dispelling myths and taking action. Disasters.

[2] Spiegel P, Rygaard Bennedsen A, Claass J, Bruns L, Patterson N, Yiweza D, Schilperoord M (2007). Prevalence of HIV infection in conflict-affected and displaced people in seven sub-Saharan African countries: a systematic review. Lancet.

[3] Mills EJ, Singh S, Nelson BD, Nachega JB (2006). The impact of conflict on HIV/AIDS in sub-Saharan Africa. Int J STD AIDS.

[4] Donovan P (2002). Rape and HIV/AIDS in Rwanda. Lancet.

[5] Jewkes R (2007). Comprehensive response to rape needed in conflict settings. Lancet.

[6] MSF. Out of Focus: How Will Millions of People in West and Central Africa are Being Left Out of the Global HIV Response. 2016. https://www.msf.org/sites/msf.org/files/2016_04_hiv_report_eng.pdf. Accessed 2 Mar 2018.

[7] WHO. Maternal Mortality Key Facts. 2018 http://www.who.int/news-room/fact-sheets/detail/maternal-mortality. Access 6 Aug 2018.

[8] UNAIDS/PCB (36)/15.13 (2015). HIV in emergency contexts: background note for UNAIDS Programme Coordinating Board, 36th meeting.

[9] WFP. El Nino Global Snapshot. 2016. https://reliefweb.int/sites/reliefweb.int/files/resources/WFP%20El%20Nino%20Global%20Snapshot%20-%20April.pdf. Accessed 6 Aug 2018.

[10] WHO.HIV and Adolescents: Guidance for HIV Testing and Counselling and Care for Adolescents Living with HIV: Recommendations for a Public Health Approach and Considerations for Policy-Makers and Managers. 2013. https://www.ncbi.nlm.nih.gov/books/NBK217964/ Accessed 6 Aug 2018. 25032477

[11] UNAIDS 2001 Declaration of Commitment on HIV/AIDS 2001. http://www.unaids.org/en/aboutunaids/unitednationsdeclarationsandgoals/2001declarationofcommitmentonhivaids. Accessed 2 Mar 2001.

[12] WHO, Strategic Framework for Emergency Preparedness. 2017. http://apps.who.int/iris/bitstream/10665/254883/1/9789241511827-eng.pdf. Accessed 2 Mar 2018.

[13] UNISDR. Build Back Better in Recovery, Rehabilitation and Reconstruction. 2017. https://www.unisdr.org/we/inform/publications/53213. Accessed 2 Mar 2018

[14] Lo STT, Chan EYY, Chan GKW (2017). Health emergency and disaster risk management: developing the research field within the Sendai framework paradigm. Int J Disaster Risk Sci.

[15] UN Office for the Coordination of Humanitarian Affairs (OCHA), International Organization on Migration (IOM). The Grand Bargain - A Shared Commitment to Better Serve People in Need. 2016. https://reliefweb.int/report/world/grand-bargain-shared-commitment-better-serve-people-need Accessed 6 Aug 2018.

[16] WHO. Bangkok Principles for the implementation of the health aspects. 2016. http://www.who.int/hac/events/2016/Bangkok_Principles.pdf. Accessed 3 Mar 2018.

[17] FEMA. National Disaster Recovery Framework (& 2nd version). 2016 https://www.fema.gov/media-library-data/1466014998123-4bec8550930f774269e0c5968b120ba2/National_Disaster_Recovery_Framework2nd.pdf. Accessed 3 March 2018].

[18] IASC. Reference Module for Cluster Coordination at Country Level. 2015. https://interagencystandingcommittee.org/iasc-transformative-agenda/documents-public/reference-module-cluster-coordination-country-level. Accessed 3 Mar 2018.

[19] GFDRR, World Bank, UNDP. Guide to Developing Disaster Friendly Frameworks. 2015. https://www.gfdrr.org/en/Guide-to-Developing-Disaster-Recovery-Frameworks. Accessed 3 Mar 2018.

[20] UNISDR. The Sendai Framework for Disaster Risk Reduction (2015–2030). 2015. https://www.unisdr.org/we/inform/publications/43291. Accessed 2 Mar 2018.

[21] UNISDR. Post 2015 Framework for Disaster Risk Reduction 2014. https://www.unisdr.org/we/inform/publications/35070. Accessed 3 Mar 2018.

[22] ODI. Disaster Resilience for Sustainable Development. 2014. https://www.odi.org/sites/odi.org.uk/files/odi-assets/publications-opinion-files/9248.pdf. Accessed Mar 3 2018.

[23] Inter-Agency Standing Committee (IASC). IASC Policy on Protection in Humanitarian Action. 2013. https://fscluster.org/sites/default/files/documents/iasc_policy_on_protection_in_humanitarian_action1.pdf. Accessed Mar 3 2018.

[24] Inter-Agency Standing Committee (IASC). IASC Task Team on Accountability to Affected Populations and Protection from Sexual Exploitation and Abuse (AAP/PSEA). 2012. https://interagencystandingcommittee.org/accountability-affected-populations-including-protection-sexual-exploitation-and-abuse. Accessed Mar 3 2018.

[25] Inter-Agency Standing Committee (IASC). IASC Transformative Agenda. 2013. https://interagencystandingcommittee.org/iasc-transformative-agenda. Accessed 3 Mar 2018.

[26] FEMA. National Response Framework. 2011. https://www.fema.gov/pdf/recoveryframework/ndrf.pdf. Accessed 3 Mar 2018.

[27] UNICEF and UNHCR. HIV/AIDS, Conflict and Displacement Conference Report. 2006. Accessed on 6 Aug 2018. http://data.unaids.org/pub/report/2006/hiv_aids_conflict_displacement.pdf

[28] UNISDR. The Hyogo Framework for Action 2005–2015: Building the Resilience of Nations and Communities for Disasters. 2005. https://www.unisdr.org/we/inform/publications/1037. Accessed 3 Mar 2018.

[29] UNISDR. 1999. The International Strategy for Disaster Reduction: A Safer World in the 21st Century: Disaster and Risk Reduction. http://www.eird.org/eng/revista/No15_99/pagina2.htm. Accessed 2 Mar 2013.

[30] UNISDR. The Yokohama Strategy for a Safer World. 2014. https://www.unisdr.org/we/inform/publications/8241. Accessed 3 Mar 2018.

[31] UNISDR. The International Framework of Action for the International Decade for Natural Disaster Reduction. 1989. https://www.unisdr.org/we/inform/publications/31468. Accessed 2 Mar 2018.

[32] Inter-agency Standing Committee. Guidelines for HIV AIDS Interventions in Emergency Settings. 2004. http://data.unaids.org/publications/external-documents/iasc_guidelines-emergency-settings_en.pdf Accessed 6 Aug 2018.

[33] Sphere Minimum Standards. Standards in Humanitarian Response. 2011. http://www.sphereproject.org/handbook/. Accessed 6 Aug 2018.

[34] InterAgency Working Group on Reproductive Health in Crises.Minimum Initial Service Package for Reproductive Health. 2011. http://iawg.net/minimum-initial-service-package/. Accessed 6 Aug 2018.

[35] World Health Organization (WHO). HIV/AIDS Guidelines in Emergencies. 2007. http://www.who.int/hac/techguidance/pht/HIV_AIDS_101106_arvemergencies.pdf?ua=1. Access 6 Aug 2018.

[36] Inter-agency Standing Committee (IASC). Guidelines on Mental Health and Psychosocial Support in Emergency Settings. 2007. http://www.who.int/mental_health/emergencies/guidelines_iasc_mental_health_psychosocial_june_2007.pdf. Accessed 6 Aug 2018. 10.1080/09540261.2022.2147420 36502397

[37] Inter-Agency Standing Committee (IASC) Reference Group on Gender and Humanitarian Action. Guidelines for Gender-based Violence Interventions in Humanitarian Settings. 2015. https://gbvguidelines.org/wp/wp-content/uploads/2015/09/2015-IASC-Gender-based-Violence-Guidelines_lo-res.pdf. Accessed Aug 6 2018.

[38] Inter-Agency Standing Committee (IASC) Reference Group on Gender and Humanitarian Action. Gender Handbook for Humanitarian Action. 2017. https://interagencystandingcommittee.org/gender-and-humanitarian-action/content/iasc-2017-gender-handbook-humanitarian-action-english. Accessed 6 Aug 2018.

[39] Inter-agency Standing Committee (IASC). Health Cluster Guide: A practical guide for country-level implementation of the Health Cluster. 2009. https://www.who.int/hac/network/global_health_cluster/health_cluster_guide_6apr2010_en_web.pdf. Accessed Mar 2 2018.;

[40] Inter-agency Standing Committee (IASC). Health Resources Availability Mapping System (HeRAMS), 2009. https://www.who.int/hac/network/global_health_cluster/herams_services_checklist_eng.pdf. Accessed Mar 2 2018.

[41] UN World Health Organization (WHO), Clinical Management of Survivors of Rape. A Guide to the Development of Protocols for Use in Refugee and Internally Displaced Person Situations. 2005. http://www.refworld.org/docid/403b79a07.html. Accessed 2 Mar 2018.

[42] Roka JL (2014). One size fits all? Standardised provision of care for survivors of sexualviolence in conflict and post-conflict areas in the Democratic Republic of Congo. PLoS ONE.

[43] Interagency Task Team to address HIV in Humanitarian Emergencies. PMTCT in Humanitarian Settings. 2015. https://www.childrenandaids.org/sites/default/files/2017-04/IATT_Part-1-PMTCT-in-Humanitarian-Settings_2015.pdf. 2 Mar 2018.

[44] O'Brien DP (2009). Universal access: the benefits and challenges in bringing integrated HIV care to isolated and conflict affected populations in the Republic of Congo. Confl Heal.

[45] US Agency International Development (USAID). October 2015 Presentation, five minutes with an expert. State of the Art Meeting.

[46] London School of Hygiene and Tropical Medicine, the Harvard School of Public Health and the Overseas Development Institute. An evidence review of research on health interventions in humanitarian crises. 2015. http://www.elrha.org/wp-content/uploads/2015/01/Evidence-Review-22.10.15.pdf. Accessed 6 Aug 2018.

