# Supplementary material for: Prioritizing the sexual and reproductive health and rights of adolescent girls and young women within HIV treatment and care services in emergency settings: a girl-centered agenda
Source: Reprod Health. 2019 May 29;16(Suppl 1):57. doi: 10.1186/s12978-019-0710-0 (PMC6538549; doi:10.1186/s12978-019-0710-0)
Supplement: Supplementary file 1 — Country Snapshots of HIV Service Gaps and Opportunities in Emergency Setting. (PDF 58 kb) [file 12978_2019_710_MOESM1_ESM.pdf]

Figure I: Country Snapshots of HIV Service Gaps and Opportunities in Emergency Setting

Rapid Onset Disasters

Cote d'Ivoire

The onset of violence Côte d'Ivoire following a failure to resolve its disputed 2010 election results led to the deaths of thousands of individuals Supporters of incumbent President Laurent Gbagbo and challenger Alassane Ouattara battled for control of the country, resulting in the deaths of thousands of civilians [47]. According to UNHCR, more than 500,000 Ivoirians were forcibly displaced, and 94,000 Ivoirians fled to neighboring Liberia out of fear of violence [48]. UNAIDS estimated there were more than 70,000 Ivoirians on ART [49]. During this period, efforts to ensure continuous access to ART materialized through: use of radio announcements to inform displaced persons of central clinics providing services; negotiations on drug delivery brokered by Ministries of Health and Defence with military and rebel groups to remove blockages: revisions and directives for screening and treatment of HIV patients and prioritizing increased months (from one to two) of supply of medicines in Bouake. The Directorate for Community Health, Information, Planning and Evaluation, and the National Public Health Pharmacy in addition to health providers and lastly members of the community support group with support from AIDS Support and Technical Resources partners, were instrumental in assisting displaced HIV patients during the crises [50]. A National Contingency Plan was developed prior to the election, which was a joint effort between US Government/PEPFAR, UN, Global Fund, and the Ministry of Health [50].

Haiti

Haiti was struck by a 6.7 magnitude earthquake on January 12, 2010 which led to about 220,000 deaths and displaced 1.5 M persons. Haiti has suffered from a series of natural disasters, including four hurricanes within a 30-day period in 2008, tropical storm Jeanne which caused massive flooding, displaced populations, in 2004, as well as Hurricane Matthew in 2016 [51]. Even years after the earthquakes, a number of camps remained in existence, forcing adolescents to survive in situations that undermine their sexual and reproductive health and expose them to risks of contracting HIV. The tripartite phenomena of vulnerability during and post-emergency, and to a great extent before the recurring natural disasters in Haiti, similar to other countries in this review, has spawned the development of several Calls to Action by the United Nations Children's Education Fund (UNICEF) over the past decade in the form of discussion papers, advocacy and recommendations. One key recommendation is for deeper consideration of adolescents and young girls living with HIV, specifically in program planning by the United Nations health cluster system. A sub set of clusters such as Gender, Health, Education and Child Protection, were identified as well-positioned to cover more detailed technical guidance on HIV and adolescent sexual and reproductive health service delivery including community, health provider and peer-led mobilization so more adolescents.

Protracted Crises

Nigeria

Several years of conflict perpetuated by Boko Haram and Islamic State of Iraq and Syria–West Africa have triggered a humanitarian crisis in Nigeria [52]. The UN estimates that nearly 11 million people in the region require humanitarian assistance, including approximately 8.5 million people in northeastern Nigeria's three most-affected states—Adamawa, Borno, and Yobe. Nigeria is Africa's most populous nation and is home to the world's third largest population of persons living with HIV [52]. Physical safety coupled with access to services for AGYW, hinges on child protection and related HIV treatment programming that often includes accounting for mobility. These realities warrant a comprehensive multi-sectoral approach to address health status of young girls specifically, entangled in war and those that find themselves as prisoners and returnees. The abductions of over 100 school girls in Northeastern Nigeria in February 2018 and over 250 in April 2014 covered in national and international news, suggests an interplay and tensions between resources allocations for military operations, counterterrorism efforts as well support for girls and families affected, in these situations.

Democratic Republic of the Congo

According to the United Nations, approximately 4.1 million people were internally displaced in DRC as of November 2017, including 1.7 million—or 41 percent of the total—newly displaced between January and October [53].The Democratic Republic of the Congo has dealt with a protracted conflict, following three civil wars and ongoing unrest between 1993 and 2003 [54]. Despite the implementation of a peace agreement in 2003, fighting between forces loyal to the Government of DRC and various armed entities, including the Allied Democratic Forces, Mai-Mai militants, and the Democratic Forces for the Liberation of Rwanda, has contributed to high levels of insecurity and population displacement in eastern DRC [55]. Violence, restricted humanitarian access, poor infrastructure, forced recruitment into armed groups, and reduced access to agricultural land and traditional markets have contributed to the deterioration of humanitarian conditions in DRC and triggered mass internal displacement and refugee outflows. Intermittent conflict continues in several eastern provinces, and new clashes are occurring in the west. These conflicts have disrupted DRC's already limited health care infrastructure. UNAIDS estimates there are 370,000 people living with HIV in the DRC [55]. A rapid assessment that was conducted during the recent conflict in the Kasai region identified Prevention of Mother to Child Transmission of HIV (PMTCT), as one of the biggest gaps in accessing HIV services.

South Sudan

The humanitarian crisis in South Sudan continues to worsen according to the UN Office of Humanitarian Coordination, which estimates that there are 7 million in need of assistance. The number of displaced people uprooted since the start of the conflict in 2013 has reached more than 4 million, including 1.9 million internally displaced people, with up to 85 per cent estimated to be children and women [56]. More than 2 million people have departed to neighboring countries—up 1.3 million since the renewed violence in July 2016 [56]. Recent

work undertaken and described by International Organization of Migration and partners, at Bentiu, Malakal, and Wau protection of civilian (PoC) sites, included HIV testing and treatment for those diagnosed positive, prevention of mother to child transmission and training of peer counselors to support clients access to and uptake of services. The shift from a predominately HIV-centered suite of services for IDPs to extending services to the general population currently visiting the clinics, implies that stigma associated with HIV or being "sick" in these three sites may be on the decline. One area for further exploration is the extent to which adults in comparison to adolescent data can be disaggregated, especially for those under 16 years of age given the wide practice of early and intergenerational marriage in South Sudan. Understanding and documenting adolescent-friendly approaches and/or strategies deployed for young girls or first time mothers, as a proportion of testing, treatment or other HIV care and support activities, remain important and relevant for all conflict settings.

References

47. The Crisis in Cote d'Ivoire. 2010. http://www.responsibilitytoprotect.org/index.php/crises/crisis-in-ivory-coast. Accessed 2 Mar 2018.

48. UNHCR. Cote d'Ivoire COI Compilations. 2017. https://data2.unhcr.org/en/documents/download/60075. Accessed 20 Feb 2018.

49. UNAIDS. Cote d'Ivoire. 2011. http://www.unaids.org/en/regionscountries/countries/ctedivoire. Accessed 20 Feb 2018.

50. AIDSTAR One Case Study. Emergency Planning for HIV Treatment in Conflict Settings. 2014. https://aidsfree.usaid.gov/sites/default/files/aidstar-one_ciemergplan.pdf. Accessed 2 Mar. 2018.

51. Malow R, Rosenberg R, Lichtenstein B, Dévieux JG. The impact of disaster on HIV in Haiti and priority areas related to the Haitian crisis. JANAC. 2010;21(3):283-288. https://doi.org/10.1016/j.jana.2010.02.002.

52. USAID. Lake Chad Basin Complex Emergency Fact Sheet. 2018. https://www.usaid.gov/crisis/lake-chad/fy18/fs7. Accessed 2 Mar. 2018.

53. USAID. Democratic Republic of the Congo Complex Emergency Fact Sheet #1 Jan 2018. https://reliefweb.int/sites/reliefweb.int/files/resources/12.11.17%20-%20USAID-DCHA%20DRC%20Complex%20Emergency%20Fact%20Sheet%20%231.pdf. Accessed 2 Mar. 2018.

54. UNAIDS. DRC. http://aidsinfo.unaids.org/. Accessed 2 Mar 2018.

55. UN OCHA. Crisis Overview South Sudan. 2018. https://reliefweb.int/sites/reliefweb.int/files/resources/South_Sudan_2018_Humanitarian_Needs_Overview.pdf. Accessed 2 Mar. 2018.

56. UN News. UN migration agency expands HIV/AIDS services in South Sudan displacement sites. 2018. http://www.un.org/apps/news/story.asp?NewsID=58057#.WgSB4PlSyM8. Accessed 2 March 2018.
