# Supplementary material for: Prioritizing the sexual and reproductive health and rights of adolescent girls and young women within HIV treatment and care services in emergency settings: a girl-centered agenda
Source: Reprod Health. 2019 May 29;16(Suppl 1):57. doi: 10.1186/s12978-019-0710-0 (PMC6538549; doi:10.1186/s12978-019-0710-0)
Supplement: Supplementary file 2 — Translation of this article into French. (PDF 312 kb) [file 12978_2019_710_MOESM2_ESM.pdf]

Priorisation de la santé sexuelle et reproductive et des droits des adolescentes et jeunes femmes au sein d'un traitement anti-VIH et de services de soins dans les situations de crise : un programme centré sur les filles

Uchechi Roxo[1*] , M. Linda Mobula[2], Damilola Walker[3], Allison Ficht[1], Sarah Yeiser[1],

USAID, 2100 Crystal Drive, Arlington, VA, USA[1]
USAID, 1300 Pennsylvania Ave NW, Washington, DC, USA[2]
UNICEF, 3 UN Plaza, New York City, New York, USA[3]

MLM: mmobula@usaid.gov
DW: dwalker@unicef.org
AF: aficht@usaid.gov
SY: syeiser@usaid.gov

*Auteur-ressource : Uchechi Roxo, uroxo@usaid.gov

Résumé

Contexte : Il existe une documentation abondante présentant des statistiques négatives sur la santé sexuelle et reproductive en termes de violations des droits durant des situations de crise humanitaire. Nous explorons deux questions centrales : les politiques, services et recherches existants abordent-ils de manière adéquate les droits de santé sexuelle et reproductive, les priorités et les risques de VIH chez les adolescentes et les jeunes femmes dans les situations de crise ? Quelles sont les occasions manquées de traiter intégralement les vulnérabilités subies par celles qui vivent avec le VIH durant des catastrophes brusques et durant des urgences prolongées sur le long terme ? Les auteurs passent en revue des considérations soutenant la prise de décisions en temps réel et soulignent les occasions manquées d'appliquer une mise en lumière genrée dans la fourniture de services liés aux droits de santé sexuelle et reproductive et au VIH, centrés sur les adolescentes et les jeunes femmes.

Méthodes : Une analyse de portée a identifié des études sur les interventions et résultats liés au VIH dans les situations de crise, publiées dans la littérature approuvée par les pairs (2002-2017). Cet exercice a été complété par un contrôle documentaire des orientations normatives, cadres et directives de mise en œuvre sur le VIH et la SSR dans les réponses d'urgence, et par des consultations auprès d'experts.

Résultats : Les cadres et orientations existants accordent peu d'attention à la santé sexuelle et reproductive et aux droits des jeunes femmes porteuses du VIH (FPVIH), en se concentrant surtout sur la prévention de la transmission de la mère à l'enfant (PTME), le traitement antirétroviral (TAR), les services de dépistage du VIH et le lien avec les services de traitement. Appliquer une mise en lumière genrée sur la santé sexuelle et reproductive offre des opportunités pour identifier les questions critiques sur l'implémentation et mettre en exergue des pratiques prometteuses, afin de mieux adapter les services existants aux AJF.

Conclusions : Une multitude de besoins concurrentiels occupe le temps et l'espace qu'on devrait dédier à l'intégration efficace des interventions sur le VIH et la santé sexuelle et reproductive dans les situations de crise. Une volonté politique est nécessaire pour faire avancer la coopération multisectorielle, grâce à une planification conjointe, un apprentissage doublé d'une information sur les droits et des réponses intégratives, et pour promouvoir des solutions créatives visant à la poursuite des TAR, l'approvisionnement en médicaments et en tests de dépistage du VIH, les traitements et les soins. Les dernières avancées en termes de politique et de pratique suggèrent qu'une réponse plus centrée sur les AJF est possible.

Mots clés

VIH/SIDA, adolescentes et jeunes femmes, humanitaire, conflit, crise, catastrophe, cadre, urgence, TAR, santé sexuelle et reproductive, directives, droits

Contexte

Les urgences humanitaires ont parfois perturbé la fourniture de services contre le virus de l'immunodéficience humaine (VIH), compliquant ainsi la traçabilité des patients prenant des traitements antirétroviraux (TAR), le maintien et l'accès aux dossiers de soins, la continuité des services et des chaînes d'approvisionnement, et entraînant même la délocalisation des soignants. Tout ceci tend à se produire dans des systèmes de santé ayant déjà pu présenter des déficits [1]. Un autre obstacle à la réponse globale apportée au VIH est que certaines populations touchées sont rendues plus vulnérables à l'infection par le VIH en raison de déplacements, de l'insécurité alimentaire et de la pauvreté, qui peuvent persister longtemps après des périodes de crise [2].

Comme expliqué dans la littérature, dans de nombreux contextes d'urgence, les femmes et les filles sont victimes de graves violations des droits de l'Homme provenant d'une inégalité des sexes enracinée, incluant un accès limité à la prévention du VIH et aux services de santé reproductive, l'exposition professionnelle forcée, le viol comme arme de guerre, le trafic, les relations sexuelles imposées, rémunérées et/ou visant à subvenir à ses propres besoins et à ceux des personnes à charge [1,3-6].

Plus la fréquence et la durée des urgences humanitaires augmentent, plus les conséquences sont dramatiques pour les femmes et les filles. Selon l'Organisation mondiale de la Santé (OMS), plus de la moitié des décès maternels se produisent dans des situations de fragilité et de crise humanitaire [7]. Selon les estimations d'ONUSIDA, plus de 1,6 million de personnes

porteuses du VIH ont été affectées par des contextes de crise humanitaire en 2013, dont 60 % de femmes [8]. Dans la sécheresse induite en 2017-2018 par El Nino, on estime à 32 millions le nombre de personnes ayant subi une insécurité alimentaire dans les pays les plus durement touchés de l'Afrique de l'est et australe, où plus de 50 % des porteurs du VIH vivent [9,10].

Admettant les trajectoires uniques par lesquelles les catastrophes humanitaires compliquent la réponse globale, une Déclaration d'engagement sur le VIH/SIDA en juin 2011 a été adoptée lors de la Session extraordinaire de l'Assemblée générale des Nations Unies (UNGASS) sur le VIH/SIDA, stipulant que “les populations déstabilisées par les conflits armés... en particulier les réfugiés et les personnes déplacées, et notamment les femmes et les enfants, sont plus exposées au risque d’infection par le VIH” [11].

Une étude sommaire des manuscrits, cadres et directives existants fait valoir que les adolescentes et les jeunes femmes (AJF) infectées par le VIH comptent parmi les plus vulnérables dans les contextes d'urgence. Cependant, il y a peu de conseils structurant une approche intégrée et sensible au développement sur les soins et traitements liés au VIH, et sur la santé sexuelle et reproductive (SSR). Alors que les parties prenantes peuvent être capables d'utiliser les sources reconnues mondialement, telles que Le Plan d'urgence présidentiel américain pour la lutte contre le SIDA (PEPFAR), le Comité permanent interorganisations (IASC), la Task Force for HIV/AIDS, le réseau SPHERE et le Programme commun des Nations Unies sur le VIH/SIDA (ONUSIDA) pour s'orienter dans la fourniture de services, leur modification pour les adapter aux environnements locaux affectés par des urgences est une véritable terra incognita dans les pays explorés par cette étude. Même les modèles de prévention, de soins et de traitements les plus solides sur le VIH requièrent une simplification et une adaptation dans un état de crise ou d'urgence, et les révisions ou suppléments spéciaux pour les pays durant des urgences restent lacunaires.

L'objectif de ce papier est d'examiner les politiques existantes et les approches de mise en œuvre identifiées par une analyse de portée en lien avec la fourniture de services concernant le VIH et la santé reproductive pour les AJF, et de faire émerger des recommandations qui pourraient transformer le paradigme actuel. Cet examen pointe aussi des suggestions d'experts dont les parties prenantes peuvent s'inspirer pour accroître la réponse aux besoins et aux vulnérabilités dans la prise en charge de ce groupe.

Méthodes :

Sélection d'études

Entre juin 2017 et décembre 2017, nous avons parcouru PubMed, Embase, le Cumulative Index of Nursing and Allied Health Literature (CINAHL) et Google Scholar à la recherche d'études qui évaluent si les normes et pratiques actuelles de réponse aux crises sont adaptées aux risques, vulnérabilités et besoins en développement spécifiques aux adolescentes et aux jeunes femmes (10 à 24 ans) porteuses du VIH. Mots clés pour la recherche : VIH et adolescentes et aide humanitaire ; médecine humanitaire ; (VIH pédiatrique) ET (conflit OU humanitaire OU crise OU urgence) ; enfant ou jeune et VIH et (crise ou conflit) ; ((adolescente) ET VIH) ET (urgence OU conflit OU crise OU humanitaire) ; ((((VIH) ET conflit) OU catastrophe) OU crise) OU humanitaire) ; ((soins VIH) ET enceinte) ET (humanitaire OU crise OU urgence OU conflit) ; ((soins VIH) ET enceinte) ET (humanitaire OU crise OU urgence OU conflit) ; famine et VIH.

Nous avons généré une base de données pour documenter les études potentiellement pertinentes. Pour identifier d'autres études, que nous pourrions avoir manquées, nous avons passé en revue les listes de références des articles sélectionnés dans notre recherche primaire.

Nous avons inclus des essais contrôlés randomisés (ECR), des études de cohortes transversales, d'observation, rétrospectives, avant/après et qualitatives. Les études qualitatives ont été incluses dès lors qu'elles étaient basées sur la collecte de données primaires. Les études systématiques et rétrospectives ont été analysées si elles comportaient des informations sur le recoupement des SSR à travers la cascade clinique pour les AJF. Nous avons aussi conduit une analyse secondaire des politiques nationales, cadres mondiaux et directives internationales en provenance de pays très touchés par le VIH qui ont connu des urgences significatives lors de la période d'étude, pour accentuer le processus de priorisation.

Les critères d'inclusion suivants ont été appliqués pour sélectionner les études : données primaires ou secondaires sur la prévention du VIH/la santé sexuelle, résultats cliniques ou psychosociaux ; rapportées à une intervention clinique, de politique, juridique ou programmatique ; rapportées aux résultats dans la population au sein d'un contexte de programme d'urgence (déclenchement rapide, déclenchement lent, et/ou urgences complexes prolongées à long terme) ; ou adolescentes, jeunes femmes ou femmes enceintes de 10 à 24 ans clairement incluses comme population étudiée.

<u>Extraction des données et évaluation de la qualité</u>

Les résumés ont été analysés par deux personnes indépendantes, qui ont inclus des articles répondant aux critères d'inclusion susmentionnés. Une analyse indépendante de la sélection d'études primaires a été effectuée par au moins un autre examinateur. Les écarts ont été résolus par consensus. Une recherche manuelle indépendante, essentiellement mais pas exclusivement à partir de références de manuscrits, a identifié des rapports supplémentaires à inclure. Les études potentiellement redondantes ont été signalées pour analyse et résolution par consensus. L'analyse intégrale des textes par toute l'équipe d'étude a été nécessaire pour établir un consensus quant à l'inclusion des rapports sur lesquels existait un doute.

L'abstraction des données a été effectuée par cinq chercheurs indépendants qui ont résumé dans des tableaux standardisés les informations sur les populations, les interventions, les comparateurs, les résultats, et le contexte/la situation. Une analyse indépendante de l'abstraction des données a été effectuée par au moins un autre chercheur.

Synthèse et analyse des données

Nous avons examiné si les normes et pratiques actuelles de réponse aux crises étaient adaptées aux risques, vulnérabilités et besoins en développement spécifiques aux adolescentes et aux jeunes femmes (10 à 24 ans) porteuses du VIH.

Consultations post-analyse : Nous avons consulté des experts de terrain, pour obtenir une opinion experte sur l'implémentation des programmes et des expériences de coordination des parties prenantes avec les programmes AJF, VIH et SSR dans les situations de crise, sur la base du travail actuel ou passé en République démocratique du Congo, Sud Soudan, Côte d'Ivoire, Nigeria et/ou dans toute l'Afrique de l'ouest et centrale. Ces consultations ont fourni des perspectives supplémentaires, vu la rareté de la littérature contrôlée par les pairs sur cette thématique, et a mis en exergue des zones qui mériteraient un dialogue et des investigations plus poussés. Ces consultations devaient nous permettre de répondre aux interrogations suivantes :

1. Quelles sont les modifications de priorités les plus critiques pour assurer la continuité des soins et du traitement anti-VIH dans les situations de crise ?
2. Quels sont les principaux obstacles à la fourniture de services de SSR pour les AJF ?
3. Quelles sont les priorités et opportunités pour renforcer la SSR des AJF porteuses du VIH et pour celles les plus à risque ?
4. Quelles sont les approches, les interventions ou modèles de services prometteurs ou efficaces pour intégrer la SSR dans les programmes de lutte contre le VIH dans les situations de crise humanitaire ?
5. Quels sont les acteurs, en particulier les organismes régionaux et/ou internationaux, les mieux placés pour faire avancer le programme d'intégration VIH-SSR pour mieux servir les AJF dans les zones de conflit ?

Résultats

Analyse de portée des modèles de fourniture de services anti-VIH pendant les situations de crise

La recherche a donné 1 250 articles, dont 26 étaient conformes aux critères d'inclusion de cette étude. **Le tableau 1** fournit un résumé des caractéristiques de ces études, et le **Tableau 2** résume les informations sur les Populations, Concepts/Résultats et Contexte/Situation pour les 26 études. Nous présumons que les situations de crise incluent un large éventail de situations, telles que les catastrophes à déclenchement rapide ou lent, les crises complexes prolongées, qui n'incluent pas un conflit. Dans notre analyse, nous avons classé le conflit comme un type

spécifique d'urgence, observant qu'il devient la cause la plus fréquente de crises prolongées qui pourraient empêcher la fourniture de services SSR pour les PVVIH.

On manque de preuves sur le recoupement des services de santé sexuelle et reproductive et les services VIH pour les AJF dans les contextes d'urgence. Aucun article n'a évalué des interventions spécifiques - on a plutôt vu une combinaison d'articles, études de cas et commentaires.

Vu le risque accru de violences sexuelles, d'infections sexuellement transmissibles (IST), d'avortements et fausses couches, d'accouchements prématurés, d'enfants morts-nés, de complications liées à l'accouchement, et de mortalité infantile et maternelle, on manque une occasion d'effectuer un examen croisé de ces questions dans les situations de crise affectées par une prévalence élevée du VIH ou une faible couverture par les interventions contre le VIH et/ou avec les RSSR.

Étude secondaire des poliltiques existantes, directives normatives et normes mondiales

Le contenu des principaux cadres mondiaux, des directives normatives et des normes mondiales pour l'action d'urgence et/ou liée à une catastrophe, la réponse et le rétablissement, a été examiné pour voir clairement l'orientation existante sur la prise de décisions dans les environnements d'urgence/de post-urgence et les environnements aux ressources limitées. **Le tableau 3** résume les constatations.

Nous avons identifié 20 cadres mondiaux traitant de la réponse et du rétablissement dans les situations d'urgence. Ces cadres se focalisaient généralement sur la planification, l'activation, la coordination, la surveillance, la gestion et le partage d'informations, en s'intéressant au besoin d'améliorer les programmes dirigés sur le nœud entre les secteurs humanitaire et de développement. Les considérations sur la programmation de réponses à la faim, la pauvreté, l'éducation, l'accès à l'eau, le logement et la gestion de l'écosystème ont été traitées sans relâche. Les auteurs ont constaté que les directives normatives sont souvent faibles sur la santé sexuelle et reproductive, le VIH et les besoins spécifiques de développement des adolescentes et des jeunes femmes. Parmi les cadres listés, aucun ne nommait explicitement le VIH ou ne donnait de recommandations, bien que certains (cinq sur les 20 identifiés) mentionnaient à la marge la santé, les soins de santé ou le système de santé.

Nous avons examiné les sources normatives établies autour desquelles les praticiens de la santé publique organisent leurs efforts contre le VIH/SIDA dans les situations d'urgence. Citons : les directives du Comité permanent interorganisations pour les interventions contre le VIH/SIDA dans les situations d'urgence [32] ; les standards minimum Sphere dans la réponse humanitaire

[33], le pack de services initial minimum pour la santé reproductive du Groupe de travail interagences sur la santé reproductive pendant les crises [34]. En plus, des directives sur les questions connexes sont incluses dans : la déclaration de consensus sur la délivrance d'antirétroviraux dans les situations d'urgence : négligée mais faisable [35] ; les directives du Comité permanent interorganisations sur la santé mentale et le soutien psychosocial dans les situations d'urgence [36] ; les directives pour des interventions contre les violences basées sur le sexe dans les situations de crise humanitaire du Groupe de référence IASC sur l'action de genre et humanitaire [37] ; et le Manuel de genres IASC 2017 pour l'action humanitaire du Groupe de référence IASC sur l'action de genre et humanitaire [38].

Le pack de services initial minimum pour la santé reproductive (MISP) est une compilation de mesures vitales, visant à prévenir et à gérer les conséquences des violences sexuelles ; à prévenir la surmortalité maternelle et infantile ; à réduire la transmission du VIH ; et à planifier des services de SR complets, dès les premiers jours d'une situation d'urgence [34]. Pour le MISP, il est essentiel de se former avant une catastrophe pour être prêt au déploiement aux niveaux local, régional et national. La formation doit se concentrer sur les interventions de SRS, VG, VIH et IST [34]. Dans un contexte d'urgence, le MISP doit être priorisé par le secteur/cluster de la santé et le Ministère de la santé (MS). Le rôle joué par le secteur/cluster de la santé dans la mise en œuvre du MISP est souligné dans les outils et directives du cluster santé de l'IASC [39, 40].

Une vigilance accordée aux besoins en prévention dans le contexte des situations d'urgence pourrait apporter le bénéfice supplémentaire de la détection d'une infection aiguë par le VIH, un besoin d'autant plus critique pour les AJF en âge de procréer. Les cadres existants identifient (parfois indirectement) le besoin spécifique de disponibilité de la prophylaxie post-exposition (PPE), et plus récemment, de la prophylaxie préexposition (PPrE). Le document de l'OMS « Gestion clinique des victimes de viol (GCV) : Développement de protocoles à adopter avec les réfugiés et les personnes déplacées dans leur propre pays - Edition révisée » fournit des protocoles et directives spécifiques sur l'utilisation de la PPE, ainsi que des traitements contre les maladies sexuellement transmissibles, sans mentionner spécifiquement les méthodes pour atteindre les AJF. La GCV est un composant essentiel du MISP et doit être incluse dans les programmes intégrés de santé et de lutte contre les VG. Les directives stipulent les conditions dans lesquelles les soignants doivent évaluer le risque élevé de VIH, sur la base de la prévalence générale du VIH et en fonction du risque connu et inconnu lié à l'auteur des faits [41].” Ces directives doivent être adaptées et amplifiées pour atteindre les AJF via des services médicaux et psychologiques.

Une étude datant de 2014 (Roka *et al.*) sur les schémas des violences sexuelles, les caractéristiques des victimes et des composantes de la réponse de Médecins Sans Frontières (MSF) à travers deux provinces (une en conflit, une en post-conflit) en République démocratique du Congo a montré que seulement 46 % des victimes (n=671) atteignaient la

clinique dans la fenêtre critique des 72 heures. Les raisons principales au retard dans la consultation des services étaient la peur, la honte et le manque de connaissances sur les traitements/services disponibles. De manière critique, le suivi des services de prévention (test VIH après report du test initial et réalisation de la PPE), était faible [42]. L'assistance psychologique, bien que fournie lors des visites d'admission et de suivi, était sous-utilisée en raison des difficultés majeures à retenir les victimes. Des messages et une éducation adaptés diffusés par la communauté et une ONG peuvent aider à apaiser ces craintes et accroître les connaissances sur les interventions disponibles, pour permettre aux victimes d'atteindre les services cruciaux.

MSF a été pionnier dans la documentation d'expériences programmatiques à grande échelle, et dans l'engagement consultatif avec les responsables de programme anti-VIH dans certains pays. Les contributions de MSF sont remarquables car elles offrent un pack de services complet pour les victimes de violences sexuelles, qui peut être très étendu dans certaines situations [42], avec :

> ...Examen médical complet incluant un examen de la zone génitale et/ou anale, offre de conseil et de dépistage du VIH et d'un test de grossesse, soins médicaux (contraception d'urgence (pour toutes les filles et femmes de 12 à 45 ans qui se présentent dans les 120 heures suivant le viol), prophylaxie des IST (IST pour toutes les victimes de viol), PPE du VIH (pour toutes les victimes de viol se présentant dans les 72 heures), vaccination contre l'hépatite B et le tétanos, soins des blessures si besoin), soutien psychologique, établissement d'un certificat médico-légal, aide médico-légale si besoin et mise en sécurité avec référence externe pour une assistance sociale dans les cas spécifiques... [42].

L'équipe de travail interinstitutions sur le VIH dans les situations d'urgence humanitaire [43] a recommandé au minimum d'assurer un approvisionnement continu en ARV pour les femmes enceintes et allaitantes porteuses du VIH et suivant un traitement à base d'ARV, et l'accès à des livraisons sûres et propres, un conseil sur l'allaitement et la prophylaxie périnatale concernant les bébés exposés au VIH.

L'équipe de travail de l'IASC sur la responsabilité envers les populations affectées (RPA) et sur la protection contre l'exploitation et les abus sexuels (PEAS) a été créée en 2012 pour promouvoir une culture de la responsabilité et de la protection contre l'exploitation et les abus sexuels à tous les niveaux du système humanitaire [24]. Elle encourage l'institutionnalisation de la RPA et de la PEAS au sein des organisations humanitaires et soutient la matérialisation de la RPA et de la PEAS au niveau collectif, ainsi qu'au niveau de l'agence individuelle, des éléments critiques des programmes réactifs auxquels les acteurs humanitaires ont été récemment sensibilisés.

Discussion

Alors que le VIH est souvent vu comme une moindre priorité dans les contextes d'urgence, en particulier en raison des autres besoins, des infrastructures de santé affaiblies et de la faible disponibilité des professionnels de santé, prévoir des cadres expérimentés qui se concentrent sur le traitement du VIH et les besoins des PVVIH peut accroître la capacité clinique et contribuer à alléger les contraintes sur les effectifs. Seconder le cluster de santé et les équipes de réponse à la catastrophe avec des spécialistes du VIH peut être bénéfique au-delà de l'étendue des services concernant le VIH apportés aux AJF. Une étude de 2002 réalisée par O'Brien *et al.* en République démocratique du Congo a montré que les « médecins qui s'occupaient des patients VIH travaillaient également dans les salles accueillant des adultes, des enfants, des urgences et des patients tuberculeux, et que des conseillers apportaient un conseil psychosocial général pour les personnes non porteuses du VIH (p. ex. conseil post-trauma) ainsi qu'un conseil lié au VIH et des activités d'éducation [44]. » D'autres médecins, conseillers et techniciens de laboratoire peuvent être inclus dans le personnel et contribuer à la formation des formateurs (FF), combler les besoins médicaux de routine et les besoins de santé physique et mentale des AJF.

Comme les services VIH sont conçus pour les AJF, et en particulier les victimes de violences sexuelles dans les contextes de crise, le suivi doit être considéré comme un aspect vital du programme. Ce soutien peut aussi prendre la forme d'une vulgarisation menée par la communauté. Dans un exemple venant du Sud Soudan, une activité DE LAVAGE centrée sur les femmes (conçue à la base pour aider les filles à risque) a accru leur vulnérabilité avec un harcèlement et des agressions sexuelles répandus et grossièrement cachés sur des sites cibles. Après que des femmes et des filles avaient rapporté des agressions sexuelles lors de leurs passages aux latrines et aux points de collecte d'eau le soir, elles se sont organisées pour des collectes en groupe et pour des réunions récurrentes, formant un système d'aide pour les victimes et les autres. Cette assemblée locale a permis aux partenaires de mettre en œuvre des services médicaux, psychosociaux et de gestion de dossiers [45]. Plus important encore, un effet d'entraînement du rassemblement des AJF a incité un sous-groupe à consulter les services de manière préventive.

L'impératif d'atteindre les AJF doit être ancré dans une évaluation rapide des besoins spécifiques, un engagement significatif des porteuses du VIH et des services post-conflit affectés, voire intégrés au besoin. L'identification et la segmentation d'une sous-population de jeunes femmes dans chaque pays d'Afrique de l'Ouest et centrale sont différentes et il est essentiel de déterminer, de développer et de délivrer les bonnes interventions et celles qui ont le plus d'impact. Par exemple, en fonction du pays, comprendre les profils des AJF et des jeunes mères est essentiel avant d'atteindre un consensus sur les interventions les plus vitales. Des

contextes variables demandent un ajustement des stratégies de délivrance des services VIH et SSR aux adolescentes au sein de la population générale, celles qui vivent avec le VIH, les primipares, les AJF professionnelles du sexe, et d'autres priorités en concurrence requérant une aide.

Comme souligné dans le MISP et les cadres de travail de l'IASC, la coordination et la communication en continu parmi les partenaires travaillant dans le secteur du VIH et de la SSR sont primordiales pour soutenir les efforts du pays hôte. Les gouvernements hôtes, le gouvernement américain/PEPFAR, le Fonds mondial de lutte contre le sida, les équipes de terrain en charge de la tuberculose et de la malaria, le coordinateur ONUSIDA, un membre de l'équipe humanitaire nationale, le cluster de santé, le Programme alimentaire mondial et enfin de nombreux exécutants financés par des donateurs et les acteurs humanitaires doivent échanger leurs informations. Avoir une ou plusieurs agences dans le rôle de coordinateur peut faciliter un rapport ponctuel et précis des tendances et modifications d'activités. Cela permet d'avoir une image des zones géographiques, d'interventions et de ressources. En plus de ces réunions des parties prenantes, les commentaires d'un directeur de bureau sanitaire régional pour l'Afrique de l'Ouest et l'Afrique centrale ont également souligné le besoin d'adapter l'assistance technique, les approches de surveillance et de supervision, et de les simplifier sur la base de ce qui est le plus faisable compte tenu de la situation d'un pays.

Les auteurs ont pointé un certain nombre de problèmes sur la base de leur prédominance à travers le contenu de l'étude. Pour la thématique mentionnée comme nécessitant une attention particulière, seuls quatre (4) articles couvraient des considérations programmatiques en lien avec les AJF, alors qu'on manquait largement d'interventions prometteuses et basées sur des preuves concernant ce groupe. La majorité des études ciblant la fourniture de services liés au VIH concerne les approches pour atteindre la population générale et réclame des solutions programmatiques alternatives lorsque ces services sont interrompus. Par conséquent, une lacune importante de ces études est le nombre de questions qui restent sans réponse -- que fait-on pour atteindre les jeunes filles et comment les AJF porteuses du VIH surmontent-elles les obstacles à la fourniture des services ; et pour celles qui recherchent habilement, reçoivent et restent en lien avec les services de traitements, de soins et de SSR dans des pays en proie à l'insécurité, quels sont les facteurs en jeu ?

Les interventions adaptées aux AJF dont le statut VIH est connu et à celles présentant un risque élevé devraient prendre en compte les victimes de viol, de violences sexuelles, les jeunes filles et femmes exploitées engagées volontairement ou de force dans le travail sexuel, ainsi que dans les transactions sexuelles. De manière spécifique, une recherche et une documentation plus poussées sont nécessaires pour savoir comment atteindre les adolescentes par des soins

basés dans un établissement ou une communauté, via leurs réseaux sociaux et sexuels, et par une délégation des tâches. L'importance d'intégrer des services SSR et VIH en Afrique de l'Ouest et centrale a été soulignée lors de forums de haut niveau, tels que la conférence internationale sur le sida en 2018 de même que les menaces à la santé des AJF et les approches pour faire avancer la prévention du VIH et la protection des enfants. Alors que le nombre d'appels à action [46] augmente pour contrer les risques, les vulnérabilités et proposer des services tenant compte du développement aux AJF porteuses du VIH dans les pays à conflits, on dispose d'une documentation limitée sur les réponses humanitaires ayant appliqué une approche tournée sur les AJF et genrée aux services liés au VIH de manière systématique en Côte d’Ivoire, à Haïti, au Nigeria, en République démocratique du Congo et au Sud Soudan -- cinq environnements en proie à des périodes de fragilité permanentes avec des implications pour la réponse du secteur sanitaire (voir **fichier additionnel 1)**
Étant donné la pauvreté de la littérature contrôlée par les pairs à ce sujet, nous avons basé notre analyse de portée sur les perspectives de praticiens possédant une expérience significative dans la coordination des services VIH et SSR dans les situations de crise. En février 2017, nous avons conduit des entretiens téléphoniques individuels avec sept experts et une réunion physique avec des organismes de santé majeurs : ONUSIDA, UNICEF, USAID West and Central Africa. Nos objectifs : découvrir les priorités principales pour renforcer les messages et les services de SSR au sein des programmes de lutte contre le VIH pour les AJF et établir des recommandations de base pour exécuter ces programmes, en s'inspirant des expériences collectives de mise en œuvre.

Tous les participants dispensaient en priorité des soins obstétriques et prénatals aux femmes enceintes porteuses du VIH ; des antirétroviraux et autres produits de base contre le VIH, une prévention contre les VG et des services aux victimes de viol. Le soutien aux AJF par la mobilisation de leur foyer et de leur communauté pour contrer le risque accru de contracter le VIH suite à un viol ou à une exploitation sexuelle a été souligné de manière répetée, tout comme la coordination des partenaires et des gouvernements hôtes.

Cinq thèmes communs de priorisation dans les situations d'urgence et les programmes adaptés aux AJF et aux femmes en âge de procréer ont été identifiés par les experts :

- Protection des femmes et des filles et réponse aux violences, y compris les questions sur les réponses efficaces et les réalités de certaines contraintes/limitations opérationnelles dans le ciblage des adolescentes et autres victimes de viol et de violences sexuelles (de même que le contrôle des conséquences inattendues et dommageables de transfert d'argent, d'espaces sécurisés et d'autres programmes)

- Grossesse et PTME, en se focalisant sur la vulnérabilité accrue des adolescentes, et approches pour garantir que les fournisseurs de services puissent répondre aux besoins de la mère et de l'enfant dont le statut VIH est connu ou inconnu
- Accès difficile au système de santé, soulignant le besoin accru de recherche sur l'accès aux contraceptifs, aux TAR et au suivi de la charge virale via des plateformes de services dans une situation de crise, tout en gérant la stigmatisation aggravée, la discrimination et les connaissances, attitudes et pratiques variées des AJF
- Soutien psychosocial et de santé mentale, en particulier besoin de plus de preuves sur les interventions individuelles les plus faisables, les plus rentables et les plus utiles comparées aux interventions de groupe.
- Expression, responsabilité et autonomisation, avec une préoccupation quant à de nouvelles modalités permettant d'amplifier et d'optimiser ces attributs pour qu'ils puissent se traduire dans des gains à travers la mobilisation communautaire, la qualité des soins et la prise en charge des AJF en cas de mobilité, de déplacement et/ou de relocalisation.

Le contenu de nos consultations a fourni des visions pratiques sur la façon de s'aligner sur les principes fondamentaux décrits dans les directives 2015 de l'équipe de travail interinstitutions sur le VIH dans les situations d'urgence humanitaire [43]. Leurs recommandations soulignent le besoin d'intégrer la SSR et les droits dans les services de lutte contre le VIH pour les AJF, mais réclament également une réponse des parties prenantes pour :

- Identifier des plates-formes adaptées (basées sur les fournisseurs, individualisées/initiées par les pairs et/ou la communauté) pour les AJF, afin de comprendre l'éventail de services (ou les plus critiques) et de contribuer à des soins et des aides au traitement sans stigmatisation. Pour les AJF touchées par la violence, améliorer l'intégration des réponses aux VG dans les programmes de santé
- Prioriser les TAR et médicaments vitaux pour les porteuses du VIH et les femmes enceintes afin d'éviter la transmission de la mère à l'enfant et renforcer les programmes transfrontaliers et les points de services mobiles qui visent à atteindre les AJF
- Renforcer l'accent porté sur la PTME pour les femmes enceintes au sein du MISP, en particulier l'accès aux options de contraception modernes pour les femmes porteuses du VIH, qui continue d'être un élément trop peu mis en avant pour assurer l'efficacité des programmes de PTME. Les acteurs de la lutte contre le VIH doivent garantir un accès continu aux services de PTME en reprogrammant rapidement leurs activités en cas de crise, tout en s'assurant que les éléments critiques du MISP sont fournis aux femmes nécessitant des services de SR ;

- Il faut communiquer des informations adaptées à la culture, à l'âge et à la langue aux filles et aux personnes qui s'en occupent, en particulier prévenir, traiter et gérer le VIH et les IST, espacer les grossesses, aborder l'hygiène menstruelle, négocier les pratiques sexuelles protégées et leur expliquer comment accéder aux services qui font partie d'un programme d'ensemble
- Mieux comprendre et répondre aux nuances variées de planification pour les populations cachées d'AJF, y compris les femmes combattantes, les travailleuses du sexe, les filles engagées dans les transactions sexuelles, les filles mariées de force et/ou mariées prématurément, les femmes dépendantes à des substances, les femmes transgenre et d'autres sous-groupes spéciaux, plutôt que supposer une homogénéité parmi les femmes cherchant ou recevant des services liés au VIH
- Soutenir les services de santé mentale dans le cadre de la diffusion et dans les contextes cliniques, y compris avec des solutions créatives qui couvrent les besoins de santé mentale. Les outiller, ainsi que d'autres fournisseurs (plus les employés) par des stratégies de formation et de transfert des tâches, ce qui permet d'étendre le pool de ressources humaines pour la santé au sein des établissements et des communautés
- S'assurer que les acteurs humanitaires, les partenaires de lutte contre le VIH et autres partenaires de santé communiquent en permanence et, si besoin, s'engagent dans une planification conjointe à travers le cluster de santé et d'autres plates-formes
- Explorer de nouvelles solutions de données et architecture à tous les niveaux, décentralisés et national, afin d'améliorer le suivi à travers les lieux et sauvegarder la sécurité des patients, y compris avec des systèmes de données mobiles, de biométrie et/ou d'information basés sur le cloud
- Mettre à jour les directives et politiques mondiales pour refléter les stratégies et mettre en œuvre des plans opérationnels spécifiques aux pays pour des services VIH et SSR intégrés dans les situations d'urgence humanitaire
- Maintenir la flexibilité des sources de financement et l'ouverture à une réorientation ou une réattribution et des ressources qui intègrent si possible la SSR et le VIH dans d'autres programmes de routine considérés comme prioritaires dans le pays concerné (p. ex., nutrition, accès à l'eau, assainissement et santé)

Conclusions

Les occasions ne manquent pas de faire avancer un programme de priorisation pour les soins centrés sur les AJF au niveau des politiques, des programmes et des informations stratégiques, afin d'améliorer la qualité et la portée des services, particulièrement dans les situations de crise. Les auteurs affirment que l'application d'une approche de SSR dans les environnements les plus exigeants est tout aussi essentielle que d'assurer une réponse multisectorielle bien

coordonnée, menée localement, afin de ne pas négliger cette population particulière. Il reste critique de définir clairement en termes pratiques des approches qui priorisent l'identification (puis la mise en relation avec les services) des adolescentes et des jeunes mères porteuses du VIH et celle les plus à risque. L'intégration d'activités des partenaires humanitaires dans la réponse au VIH peut, si elles sont centrées sur les clients, offrir la meilleure plate-forme pour atteindre les AJF dans les pays affectés. Pour aller au-delà des lacunes actuelles identifiées par l'étude de portée, les plans spécifiques aux pays, les investissements et la recherche ne peuvent pas se contenter d'un recours à des appels à action et aux récentes directives. Les exécutants doivent s'inspirer de ce qui marche dans les services de SSR dans les situations complexes de crise en Afrique, tout en apportant des nuances pour répondre aux besoins des AJF d'âges divers. La documentation rigoureuse des pratiques efficaces requiert plus d'attention, en particulier dans les situations d'urgence rapides/soudaines. Les parties prenantes doivent rester très conscientes du contexte sociologique d'une situation d'urgence particulière lorsqu'elles adaptent les interventions pour cibler les AJF dans l'accès aux services.

Liste des abréviations

RPA responsabilité envers les populations affectées
AJF adolescentes et jeunes femmes
TAR traitement antirétroviral
VG violence genrée
VIH virus de l'immunodéficience humaine
IASC Comité permanent interorganisations
GSHM gestion de la santé et de l'hygiène menstruelle
MS Ministère de la santé
MSF Médecins Sans Frontières
MISP pack de services initial minimum pour la santé reproductive
PPE prophylaxie post-exposition
PEPFAR Plan d'urgence présidentiel américain pour la lutte contre le SIDA
PTME prévention de la transmission de la mère à l'enfant
PPrE prophylaxie préexposition
PEAS protection contre l'exploitation et les abus sexuels
ECR essai contrôlé randomisé
SR santé reproductive
SSR santé sexuelle et reproductive
TB tuberculose
FF formation des formateurs
ONUSIDA Programme commun des Nations Unies sur le VIH/SIDA
UNGASS Session extraordinaire de l'Assemblée générale des Nations Unies
USAID United States Agency for International Development
OMS Organisation mondiale de la Santé

Concernant ce supplément

Cet article a été publié comme partie de l'ouvrage *Reproductive Health*, Volume 16 Supplement 1, 2019: Effective Integration of Sexual Reproductive Health and HIV Prevention, Treatment, and Care Services across sub-Saharan Africa: Where is the evidence for program implementation?
Le supplément a été publié dans le cadre d'une collaboration entre *Reproductive Health* et *BMC Public Health.* L'intégralité du contenu, avec les versions en français, en portugais et en anglais, est disponible en ligne :
https://bmcpublichealth.biomedcentral.com/articles/supplements/volume-19-supplement-1
et
https://reproductive-health-journal.biomedcentral.com/articles/supplements/volume-16-supplement-1

Déclarations

Approbation éthique et accord de participation
Non applicable

Accord de publication
Non applicable

Disponibilité des données et matériels
Non applicable

Intérêts concurrents
Les auteurs déclarent ne pas avoir d'intérêts concurrents

Financement

Le supplément de la revue est rendu possible grâce au soutien généreux du peuple américain via la United States Agency for International Development (USAID) en partenariat avec le Fonds des Nations unies pour la population (FNUAP) et le Programme commun des Nations Unies sur le VIH/SIDA (ONUSIDA).

Contributions des auteurs

UR, LM et DW ont conceptualisé les objectifs et les méthodes de l'analyse et développé la première ébauche. AF et SY ont effectué l'examen de la littérature et ébauché la section sur les

méthodes et les résultats du présent manuscrit. UR a résumé les thèmes essentiels à partir d'entretiens avec des experts clés du développement et de la santé publique. Tous les auteurs ont lu et approuvé la version finale du manuscrit.

Remerciements

Les auteurs souhaitent remercier les experts régionaux suivants en Afrique de l'Ouest et centrale pour leurs contributions, leurs réflexions basées sur leur expérience dans de nombreux pays et leur engagement inébranlable : Dr. Denis Mali, Dr. Laurent Kapesa, Dr. Charles Dago, Lekwalo Mokgulo, Sihaka Tsemo, PhD, Dr. Raoul Anderson, Maiga Modibo, Han Kangainsi que le Dr. Nicholas Baabe et Lisa Childs.

Les opinions exprimées dans la présente publication sont celles des auteurs et ne reflètent pas nécessairement les politiques officielles de l'UNICEF (United Nations Children's Fund), ni celles d'un quelconque ministère américain ni organisme public, y compris l'US Agency for International Development ou le Plan d'urgence présidentiel américain pour la lutte contre le SIDA, la mention des dénominations de ministères ou d'organismes n'implique pas non plus l'aval du gouvernement américain ou de l'UNICEF.

Informations sur les auteurs

UR est un conseiller régional à l'Office of HIV/AIDS, Global Health Bureau, US Agency for International Development (USAID). LM est un conseiller sur la santé publique et les maladies infectieuses à la Division of Preparedness, Strategic Planning and Mitigation de l'Office of U.S. Foreign Disaster Assistance (OFDA) à l'USAID. DW est conseiller cadre pour le VIH chez les adolescents à la HIV and AIDS Section, Programme Division, UNICEF New York. AF est l'analyste du Pediatric and Maternal Clinical Branch Program pour l'USAID au Global Health Bureau, Office of HIV/AIDS et SY est l'analyste de l'Integrated Services Program for USAID au Global Health Bureau, Office of HIV/AIDS

Références

1. Spiegel, Paul, HIV/AIDS among Conflict-affected and Displaced Populations: Dispelling Myths and Taking Action. Disasters, 2004, 28(3): 322–339.
2. Spiegel P, Rygaard Bennedsen A, Claass J, Bruns L, Patterson N, Yiweza D, Schilperoord M. Prevalence of HIV infection in conflict-affected and displaced people in seven sub-Saharan African countries: A systematic review. Lancet 2007; 369: 2187–2195.

3. Mills EJ, Singh S, Nelson BD, Nachega JB. The impact of conflict on HIV/AIDS in sub-Saharan Africa. Int J STD AIDS 2006; 17:713–717.
4. Donovan P. Rape and HIV/AIDS in Rwanda. Lancet 2002; 360 (Suppl):S17–S18.
5. Jewkes R. Comprehensive response to rape needed in conflict settings. Lancet 2007; 369:2140–2141.
6. MSF. Out of Focus: How Will Millions of People in West and Central Africa are Being Left Out of the Global HIV Response. 2016. https://www.msf.org/sites/msf.org/files/2016_04_hiv_report_eng.pdf. Accessed 2 Mar 2018.
7. WHO. Maternal Mortality Key Facts. 2018 http://www.who.int/news-room/fact-sheets/detail/maternal-mortality. Access 6 Aug 2018.
8. UNAIDS/PCB (36)/15.13. HIV in emergency contexts: background note for UNAIDS Programme Coordinating Board, 36th meeting. Geneva: Joint United Nations Programme on HIV/AIDS; 2015
9. WFP. El Nino Global Snapshot. 2016. https://reliefweb.int/sites/reliefweb.int/files/resources/WFP%20El%20Nino%20Global%20Snapshot%20-%20April.pdf. Accessed 6 Aug 2018.
10. WHO.HIV and Adolescents: Guidance for HIV Testing and Counselling and Care for Adolescents Living with HIV: Recommendations for a Public Health Approach and Considerations for Policy-Makers and Managers. 2013. https://www.ncbi.nlm.nih.gov/books/NBK217964/ Accessed 6 Aug 2018.
11. UNAIDS 2001 Declaration of Commitment on HIV/AIDS. 2001. http://www.unaids.org/en/aboutunaids/unitednationsdeclarationsandgoals/2001declarationofcommitmentonhivaids. Accessed 2 Mar 2001.
12. WHO, Strategic Framework for Emergency Preparedness. 2017. http://apps.who.int/iris/bitstream/10665/254883/1/9789241511827-eng.pdf. Accessed 2 Mar 2018
13. UNISDR. Build Back Better in Recovery, Rehabilitation and Reconstruction. 2017. https://www.unisdr.org/we/inform/publications/53213. Accessed 2 Mar 2018
14. Lo, S.T.T., Chan, E.Y.Y., Chan, G.K.W. et al. Health Emergency and Disaster Risk Management: Developing the Research Field within the Sendai Framework Paradigm. Int J Disaster Risk Sci.2017; 8: 145. https://doi.org/10.1007/s13753-017-0122-0
15. UN Office for the Coordination of Humanitarian Affairs (OCHA), International Organization on Migration (IOM). The Grand Bargain - A Shared Commitment to Better Serve People in Need. 2016. https://reliefweb.int/report/world/grand-bargain-shared-commitment-better-serve-people-need Accessed 6 Aug 2018:
16. WHO.Bangkok Principles for the implementation of the health aspects.2016. http://www.who.int/hac/events/2016/Bangkok_Principles.pdf. Accessed 3 Mar 2018.

17.. FEMA. National Disaster Recovery Framework (& 2nd version). 2016 https://www.fema.gov/media-library-data/1466014998123-4bec8550930f774269e0c5968b120ba2/National_Disaster_Recovery_Framework2nd.pdf. Accessed 3 March 2018]

18. IASC. Reference Module for Cluster Coordination at Country Level. 2015. https://interagencystandingcommittee.org/iasc-transformative-agenda/documents-public/reference-module-cluster-coordination-country-level. Accessed Mar 3 2018

19. GFDRR, World Bank, UNDP.Guide to Developing Disaster Friendly Frameworks. 2015. https://www.gfdrr.org/en/Guide-to-Developing-Disaster-Recovery-Frameworks. Accessed Mar 3 2018

20. UNISDR. The Sendai Framework for Disaster Risk Reduction (2015 - 2030). 2015.https://www.unisdr.org/we/inform/publications/43291. Accessed Mar 2 2018

21. UNISDR.Post 2015 Framework for Disaster Risk Reduction. 2014. https://www.unisdr.org/we/inform/publications/35070. Accessed Mar 3 2018

22. ODI. Disaster Resilience for Sustainable Development. 2014. https://www.odi.org/sites/odi.org.uk/files/odi-assets/publications-opinion-files/9248.pdf. Accessed Mar 3 2018

23. Inter-Agency Standing Committee (IASC). IASC Policy on Protection in Humanitarian Action. 2013. https://interagencystandingcommittee.org/system/files/iasc_policy_on_protection_in_humanitarian_action_0.pdf. Accessed Mar 3 2018

24. Inter-Agency Standing Committee (IASC). IASC Task Team on Accountability to Affected Populations and Protection from Sexual Exploitation and Abuse (AAP/PSEA). 2012.https://interagencystandingcommittee.org/accountability-affected-populations-including-protection-sexual-exploitation-and-abuse. Accessed Mar 3 2018

25. Inter-Agency Standing Committee (IASC). IASC Transformative Agenda. 2013. https://interagencystandingcommittee.org/iasc-transformative-agenda. Accessed Mar 3 2018

26. FEMA. National Response Framework. 2011. https://www.fema.gov/pdf/recoveryframework/ndrf.pdf. Accessed Mar 3 2018

27. UNICEF and UNHCR. HIV/AIDS, Conflict and Displacement Conference Report.2006. Accessed on 6 Aug 2018. http://data.unaids.org/pub/report/2006/hiv_aids_conflict_displacement.pdf

28.. UNISDR. The Hyogo Framework for Action 2005 - 2015: Building the Resilience of Nations and Communities for Disasters. 2005.https://www.unisdr.org/we/inform/publications/1037. Accessed mar 3 2018

29. UNISDR. 1999. The International Strategy for Disaster Reduction: A Safer World in the 21st Century: Disaster and Risk Reduction. http://www.eird.org/eng/revista/No15_99/pagina2.htm. Accessed March 2 2013

30. UNISDR. The Yokohama Strategy for a Safer World.2014.https://www.unisdr.org/we/inform/publications/8241. Accessed Mar 3 2018
31. UNISDR. The International Framework of Action for the International Decade for Natural Disaster Reduction. 1989.https://www.unisdr.org/we/inform/publications/31468. Accessed March 2 2018
32. Inter-agency Standing Committee. Guidelines for HIV AIDS Interventions in Emergency Settings.2004. http://data.unaids.org/publications/external-documents/iasc_guidelines-emergency-settings_en.pdf Accessed 6 Aug 2018
33. Sphere Minimum Standards. Standards in Humanitarian Response.2011. http://www.sphereproject.org/handbook/. Accessed 6 Aug 2018
34. InterAgency Working Group on Reproductive Health in Crises.Minimum Initial Service Package for Reproductive Health. 2011. http://iawg.net/minimum-initial-service-package/. Accessed 6 Aug 2018
35. World Health Organization (WHO). HIV/AIDS Guidelines in Emergencies. 2007. http://www.who.int/hac/techguidance/pht/HIV_AIDS_101106_arvemergencies.pdf?ua=1. Access 6 Aug 2018
36. Inter-agency Standing Committee (IASC). Guidelines on Mental Health and Psychosocial Support in Emergency Settings. 2007. http://www.who.int/mental_health/emergencies/guidelines_iasc_mental_health_psychosocial_june_2007.pdf. Accessed 6 Aug 2018
37. Inter-Agency Standing Committee (IASC) Reference Group on Gender and Humanitarian Action. Guidelines for Gender-based Violence Interventions in Humanitarian Settings. 2015. https://gbvguidelines.org/wp/wp-content/uploads/2015/09/2015-IASC-Gender-based-Violence-Guidelines_lo-res.pdf. Accessed Aug 6 2018
38. Inter-Agency Standing Committee (IASC)Reference Group on Gender and Humanitarian Action. Gender Handbook for Humanitarian Action.2017. https://interagencystandingcommittee.org/gender-and-humanitarian-action/content/iasc-2017-gender-handbook-humanitarian-action-english. Accessed 6 Aug 2018
39. Inter-agency Standing Committee (IASC), Health Cluster Guide: A practical guide for country-level implementation of the Health Cluster. 2009. www.who.int/hac/network/global_health_cluster/guide. Accessed Mar 2 2018.;
40. Inter-agency Standing Committee (IASC), Health Resources Availability Mapping System (HeRAMS), 2009. http://iawg.net/resources/Graph_RAMS_5aug09.pdf. Accessed Mar 2 2018
41. UN World Health Organization (WHO), Clinical Management of Survivors of Rape. A Guide to the Development of Protocols for Use in Refugee and Internally Displaced Person Situations. 2005.http://www.refworld.org/docid/403b79a07.html. Accessed Mar 2 2018

42. Roka, Jerlie Loko, et al. One size fits all? Standardised provision of care for survivors of sexualviolence in conflict and post-conflict areas in the Democratic Republic of Congo. PLoS ONE.2014; 9:10. doi:10.1371/journal.pone.0111096.

43. Interagency Task Team to address HIV in Humanitarian Emergencies. PMTCT in Humanitarian Settings. 2015. https://www.childrenandaids.org/sites/default/files/2017-04/IATT_Part-1-PMTCT-in-Humanitarian-Settings_2015.pdf. 2 Mar 2018.

44. O'Brien, Daniel P, et al. Universal access: the benefits and challenges in bringing integrated HIV care to isolated and conflict affected populations in the Republic of Congo. Conflict and Health 2009; 3:1. doi:10.1186/1752-1505-3-1.

45. US Agency International Development (USAID). October 2015 Presentation, Five Minutes with An Expert. State of the Art Meeting

46. London School of Hygiene and Tropical Medicine, the Harvard School of Public Health and the Overseas Development Institute. An evidence review of research on health interventions in humanitarian crises. 2015. http://www.elrha.org/wp-content/uploads/2015/01/Evidence-Review-22.10.15.pdf. Accessed 6 Aug 2018.

47. The Crisis in Cote d'Ivoire. 2010. http://www.responsibilitytoprotect.org/index.php/crises/crisis-in-ivory-coast. Accessed 2 Mar 2018.

48. UNHCR. Cote d'Ivoire COI Compilations. 2017. *https://data2.unhcr.org/en/documents/download/60075*. Accessed 20 Feb 2018

49. UNAIDS. Cote d'Ivoire. 2011. http://www.unaids.org/en/regionscountries/countries/ctedivoire. Accessed 20 Feb 2018

50. AIDSTAR One Case Study. Emergency Planning for HIV Treatment in Conflict Settings. 2014.https://aidsfree.usaid.gov/sites/default/files/aidstar-one_ciemergplan.pdf. Accessed 2 Mar. 2018.

51. Malow R, Rosenberg R, Lichtenstein B, Dévieux JG. The impact of disaster on HIV in Haiti and priority areas related to the Haitian crisis. JANAC. 2010;21(3):283-288. doi:10.1016/j.jana.2010.02.002.

52. USAID. Lake Chad Basin Complex Emergency Fact Sheet. 2018. https://www.usaid.gov/crisis/lake-chad/fy18/fs7. Accessed 2 Mar. 2018.

53. USAID. Democratic Republic of the Congo Complex Emergency Fact Sheet #1 Jan 2018. https://reliefweb.int/sites/reliefweb.int/files/resources/12.11.17%20-%20USAID-DCHA%20DRC%20Complex%20Emergency%20Fact%20Sheet%20%231.pdf. Accessed 2 Mar. 2018.

54. UNAIDS. DRC. http://aidsinfo.unaids.org/. Accessed 2 Mar 2018

55. UN OCHA. Crisis Overview South Sudan. 2018.http://www.unocha.org/country/south-sudan/crisis-overview. Accessed 2 Mar. 2018.

56. UN News. UN migration agency expands HIV/AIDS services in South Sudan displacement sites. 2018. http://www.un.org/apps/news/story.asp?NewsID=58057#.WgSB4PlSyM8. Accessed 2 March 2018

Fichiers additionnels

Fichier additionnel 1 : Country Snapshots of HIV Service Gaps and Opportunities in Emergency Setting. **Additional file 1_Roxo et al.pdf**

Tableau 1 : études conformes aux critères d'inclusion, sur la base de leur contenu thématique

	N=26
**Population**	
Adultes (uniquement)	4
Adolescents et enfants	3
Femmes (uniquement)	2
Population générale (toute PVVIH)	16
Non précisé	2
**Interventions pour les AJF abordées**	
Oui	3
Non	23
**RSSR abordées**	
Oui	9
Non	17
**Contexte**	
Conflit	13
Post-conflit	8
Conflit + post-conflit	5

Catastrophe naturelle	1
**Résultats**	
Prévention du VIH	3
Risque de VIH	2
Utilisation des services liés au VIH	15
Rétention/interruption des TAR	5
Santé reproductive	1
Mortalité imputable au VIH	1

Tableau 2 Résumé des études : classification des populations, des contextes et des résultats

**Études citées**	**Population**	**Configuration**	**Contexte**	**Résultats mesurés**	**Aborde la SSR et les AJF**
O'Brien DP, Mills C, Hamel C, Ford N, Pottie K "Universal access: the benefits and challenges in bringing integrated HIV care to isolated and conflict affected populations in the Republic of Congo." (2009)	PVVIH (adultes, adolescents, enfants)	Cohorte rétrospective	Conflit	Résultats cliniques des patients sous TAR	SSR : non<br>AJF : non

Loko Roka J, Van den Bergh R, Au S, De Plecker E, Zachariah R, Manzi M, Lambert V, Abi-Aad 1, Nanan-N'Zeth K, Nzuya S, Omba B, Shako C, MuishaBaroki D, Basimuoneye JP, Moke DA, Lampaert E, Masangu L, De Weggheleire A. "One size fits all? Standardised provision of care for survivors of sexual violence in conflict and post-conflict areas in the Democratic Republic of Congo." (2014)	PVVIH (adultes, adolescents, enfants)	Cohorte rétrospective descriptive	Conflit/post-conflit	Haut (>95 %) taux de couverture en produits prophylaxiques, avec suivi médiocre ; faibles taux de traitement et/ou de vaccination	SSR : oui AJF : oui
Atwood KA, Kennedy SB, Shamblen S, Tegli J, Garber S, Fahnbulleh PW, Korvah PM, Kolubah M, Mulbah-Kamara C, Fulton S "Impact of school-based HIV prevention program in post-conflict Liberia." (2012)	Jeunes	Étude de faisabilité utilisant un essai contrôlé randomisé	Post-conflit	Connaissances, attitude et pratique	SSR : oui AJF : oui
Holt BY, Effler P, Brady W, Friday J, Belay E, Parker K, Toole M "Planning STI/HIV prevention among refugees and mobile populations: situation	Adultes	Étude qualitative	Conflit	Connaissances, attitudes et comportements et prévalence du VIH/des IST	SSR : oui AJF : non

assessment of Sudanese refugees." (2003)					
Salami, O., A. Buzu, C. Nzeme "High Level of Adherence to HAART Among Refugees and Internally Displaced Persons on HAART in Western Equatorial Region of Southern Sudan" (2010)	PVVIH (adultes, adolescents, enfants)	Étude transversale	Conflit	Observance des TAR	SSR : non AJF : non
Patel S, Schechter MT, Sewankambo NK, Atim S, Oboya C, Kiwanuka N, Spittal PM "Comparison of HIV-related vulnerabilities between former child soldiers and children never abducted by the LRA in Northern Uganda" (2013)	Enfants	Étude transversale	Post-conflit	Prévalence du VIH	SSR : oui AJF : non
Wilhelm-Solomon M "Challenges for antiretroviral provision in northern Uganda" (2010)	PVVIH (adultes, adolescents, enfants)	Commentaires	Conflit, post-conflit	Utilisation des services liés au VIH	SSR : non AJF : non
Yoder RB, Nyandiko WM, Vreeman RC, Ayaya SO, Gisore PO, Braitstein P, Wiehe SE. "Long-term impact of the Kenya post-election	Enfants <14 ans	Cohorte rétrospective	Conflit, post-conflit	Observance des TAR	SSR : non AJF : oui

crisis on clinic attendance and medication adherence for HIV-infected children in western Kenya" (2012)					
Goodrich S, Ndege S, Kimaiyo S, Some H, Wachira J, Braitstein P, Sidle JE, Sitienei J, Owino R, Chesoli C, Gichunge C, Komen F, Ojwang C, Sang E, Siika A, Wools-Kaloustian K "Delivery of HIV care during the 2007 post-election crisis in Kenya: a case study analyzing the response of the Academic Model Providing Access to Healthcare (AMPATH) program" (2013)	PVVIH (adultes, adolescents, enfants)	Étude de cas	Post-conflit	Utilisation des services liés au VIH	SSR : non<br>AJF : non
Bamrah S, Mbithi A, Mermin JH, Boo T,Bunnell RE, Sharif S, Cookson ST "The impact of post-election violence on HIV and other clinical services and on mental health-Kenya, 2008" (2012)	Adultes	Étude descriptive	Conflit	Observance des TAR	SSR : non<br>AJF : non

Pyne-Mercier LD, John-Stewart GC, Richardson BA, Kagondu NL, Thiga J, Noshy H, Kist N, Chung MH "The consequences of post-election violence on antiretroviral HIV therapy in Kenya. AIDS Care" (2011)	Adultes	Analyse rétrospective à méthodologie mixte	Post-conflit	Observance des TAR	SSR : non AJF : non
Unge C, Södergård B, Thorson A, Ragnarsson A, Carter J, Ilako F, Waweru M, Ekström AM. "HIV treatment in times of civil strife: serious threats to antiretroviral drug access in the Kibera slum following the Kenyan elections" (2008)	Âges non spécifiés	Étude qualitative	Post-conflit	Utilisation des services liés au VIH	SSR : non AJF : non
Reid T, van Engelgem I, Telfer B, Manzi M "Lessons learned (programmatic) from MSF's three primary health care centers, including HIV treatment and support services in Kibera slum" (2008)	Âges non spécifiés	Commentaires	Post-conflit	Utilisation des services liés au VIH	SSR : non AJF : non
Anthonj C, Nkongolo OT, Schmitz P, Hango JN, Kistemann T "The impact of flooding	PVVIH (adultes, adolescents, enfants)	Étude qualitative	Catastrophe naturelle	Utilisation des services liés au VIH	SSR : non AJF : non

on people living with HIV: a case study from the Ohangwena Region, Namibia" (2015)					
Noden BH, Pearson RJ, Gomes A "Age-specific mortality patterns in Central Mozambique during and after the end of the Civil War" (2011)	PVVIH (adultes, adolescents, enfants)	Cohorte rétrospective	Conflit	Mortalité	SSR : non AJF : non
Mendelsohn JB, Schilperoord M, Spiegel P, Ross DA "Adherence to antiretroviral therapy and treatment outcomes among conflict-affected and forcibly displaced populations: a systematic review" (2012)	PVVIH (adultes, adolescents, enfants)	Analyse systématique	Conflit/post-conflit	Observance des TAR	SSR : non AJF : non
Culbert H, Tu D, O'Brien DP, Ellman T, Mills C, Ford N, Amisi T, Chan K, Venis S "HIV treatment in a conflict setting: outcomes and experiences from Bukavu, Democratic Republic of the Congo" (2007)	PVVIH (adultes, adolescents, enfants)	Étude descriptive	Conflit	Utilisation des services liés au VIH	SSR : non AJF : non

Simon, S "Review of International Federation of Red Cross and Red Crescent Societies (IFRC) material on HIV and AIDS and sudden onset emergencies" (2008)	PVVIH (adultes, adolescents, enfants)	Rapport de programme	Catastrophe naturelle	Utilisation des services liés au VIH	SSR : non AJF : non
Da Waal A, Klot J, Mahajan M "HIV/AIDS, Security and Conflict: New Realities, New Responses" (2009)	PVVIH (adultes, adolescents, enfants)	Commentaires	Post-conflit	Utilisation des services liés au VIH	SSR : oui AJF : non
Spiegel, P "HIV in emergencies – much achieved, much to do" (2010)	PVVIH (adultes, adolescents, enfants)	Commentaires	Conflit/post-conflit	Utilisation des services liés au VIH	SSR : oui AJF : non
Spiegel, P "The effects of antiretroviral therapy on HIV prevalence in conflict situations: not yet there." (2009)	PVVIH (adultes, adolescents, enfants)	Commentaires	Conflit	Utilisation des services liés au VIH	SSR : non AJF : non
Spiegel, P "Populations: Dispelling myths and taking action." (2004)	PVVIH (adultes, adolescents, enfants)	Commentaires	Conflit	Utilisation des services liés au VIH	SSR : oui AJF : non
Whitmill J, Blanton C, Doraiswamy S, Cornier N, Schilperood M, Spiegel P, Tomczyk B "Retrospective analysis of reproductive health indicators in the United	Femmes	Analyse rétrospective	Conflit	Indicateurs de santé reproductive	SSR : oui AJF : non

Nations High Commissioner for Refugees post-emergency camps 2007-2013" (2016)					
Griffiths K, Ford N "Provision of antiretroviral care to displaced populations in humanitarian settings: a systematic review." (2013)	PVVIH (adultes, adolescents, enfants)	Analyse systématique	Conflit, catastrophe naturelle	Utilisation des services liés au VIH	SSR : non AJF : non
Hankins C A "Transmission and prevention of HIV and sexually transmitted infections in war settings: Implications for current and future armed conflicts" (2002)	PVVIH (adultes, adolescents, enfants)	Commentaires	Conflit	MISP, indicateurs de planning familial de prévention du VIH, interventions pour l'injection de médicaments	SSR : oui AJF : non
Ssonko C, Gonzalez L, Mesic A, Silveira de Fonesca M, Achar J, Safar N, Martin B, Wong S, Casas E "Delivering HIV care in challenging operating environments: the MSF experience towards differentiated models of care for settings with multiple basic health care needs" (2017)	PVVIH (adultes, adolescents, enfants)	Analyse descriptive	Conflit	Utilisation des services liés au VIH	SSR : non AJF : non

**Tableau 3 :** principaux cadres mondiaux		
Année	Auteur ou organisme	Intitulé du cadre
2017	Organisation mondiale de la Santé (OMS)	Cadre stratégique de préparation aux situations d'urgence [12]
2017	Bureau des Nations Unies pour la réduction des risques de catastrophes (ONU-SIPC)	Mieux reconstruire dans les situations de redressement, de réhabilitation et de reconstruction [13]
2017	Lo, S.T.T. et al.	Health Emergency and Disaster Risk Management (Gestion des urgences sanitaires et des risques de catastrophe) : Developing the Research Field within the Sendai Framework Paradigm (développement du champ des recherches au sein du paradigme du cadre d'action de Sendai) [14]
2016	Bureau de la coordination des affaires humanitaires de l'ONU (BCAH), Organisation internationale pour les migrations (OIM)	The Grand Bargain - A Shared Commitment to Better Serve People in Need (Le grand marchandage - un engagement partagé pour mieux servir les populations dans le besoin) [15]
2016	Organisation mondiale de la Santé (OMS)	Bangkok Principles for the implementation of the health aspects (Principes de Bangkok pour la mise en œuvre des aspects sanitaires) [16]
2016 & 2013	Federal Emergency Management Agency (FEMA)	National Disaster Recovery Framework (& 2nd version) (Cadre d'action pour le rétablissement après une catastrophe d'ampleur nationale (et 2e version) [17]
2015	Comité permanent interorganisations (IASC)	Reference Module for Cluster Coordination at Country Level (Module de référence pour la coordination des clusters au niveau national) [18]
2015	Dispositif mondial de	Guide to Developing Disaster Friendly Frameworks

	réduction des effets des catastrophes et de reconstruction (GFDRR), Banque mondiale, Programme des Nations Unies pour le développement (PNUD)	(Orientation pour la mise en place de cadres d'action compatibles avec les catastrophes) [19]
2015	ONU-SIPC	Cadre d'action de Sendai pour la réduction des risques de catastrophe (2015 - 2030) [20]
2014	ONU-SIPC	Cadre d'action pour la réduction des risques de catastrophe après 2015 [21]
2014	Overseas Development Institute (ODI)	Disaster Resilience for Sustainable Development (La résilience face aux catastrophes pour un développement durable) [22]
2013	IASC	IASC Policy on Protection in Humanitarian Action (Politique de protection de l'IASC dans l'action humanitaire) [23]
2012	IASC	IASC Task Team on Accountability to Affected Populations and Protection from Sexual Exploitation and Abuse (AAP/PSEA) (équipe de travail sur la responsabilité envers les populations touchées et sur la protection contre l'exploitation et les abus sexuels) [24]
2011	IASC	IASC Transformative Agenda (programme de transformation) [25]
2011	FEMA	National Response Framework (cadre national d'action de réponse) [26]
2006	HCR & UNICEF	HIV/AIDS, Conflict and Displacement Conference Report (rapport de conférence sur le VIH/SIDA, les conflits et les déplacements) [27]
2005	ONU-SIPC	Cadre d'action de Hyogo 2005 - 2015 : Pour des nations et des collectivités résilientes face aux catastrophes [28]
1999	ONU-SIPC	Stratégie internationale pour la prévention des catastrophes : un monde plus sûr au 21e siècle :

		réduction des catastrophes et des risques [29]
1994	ONU-SIPC	La stratégie de Yokohama pour un monde plus sûr [30]
1989	ONU-SIPC	Cadre international d'action pour la Décennie internationale de la prévention des catastrophes naturelles (DIPCN) [31]
