# Supplementary material for: Prioritizing the sexual and reproductive health and rights of adolescent girls and young women within HIV treatment and care services in emergency settings: a girl-centered agenda
Source: Reprod Health. 2019 May 29;16(Suppl 1):57. doi: 10.1186/s12978-019-0710-0 (PMC6538549; doi:10.1186/s12978-019-0710-0)
Supplement: Supplementary file 3 — Translation of the abstract of this article into Portuguese. (PDF 97 kb) [file 12978_2019_710_MOESM3_ESM.pdf]

Priorização da saúde e dos direitos sexuais e reprodutivos das adolescentes e mulheres jovens nos serviços de tratamento e cuidados de VIH em cenários de emergência: um programa dedicado ao sexo feminino

Uchechi Roxo[1*], M. Linda Mobula[2], Damilola Walker[3], Allison Ficht[1], Sarah Yeiser[1],
USAID, 2100 Crystal Drive, Arlington, VA, USA[1]
USAID, 1300 Pennsylvania Ave NW, Washington, DC, USA[2]
UNICEF, 3 UN Plaza, New York City, New York, USA[3]
MLM: mmobula@usaid.gov
DW: dwalker@unicef.org
AF: aficht@usaid.gov
SY: syeiser@usaid.gov

*Autor correspondente: Uchechi Roxo, uroxo@usaid.gov

Resumo

Introdução: Existe uma vasta documentação sobre diversos resultados negativos ao nível de saúde sexual e reprodutiva e violações dos direitos humanos que ocorrem durante situações de emergência humanitária. O presente documento explora duas questões fulcrais: As políticas, a pesquisa e os serviços existentes abordam satisfatoriamente os direitos de SSR, as prioridades e os riscos de VIH das adolescentes e mulheres jovens em situações de emergência? Quais são as oportunidades perdidas para lidar de forma global com as vulnerabilidades sentidas por quem vive com o VIH durante situações de catástrofes inesperadas e emergências complexas de longa duração? Os autores analisaram as considerações sobre a tomada de decisões em tempo real e destacam as oportunidades perdidas para aplicar um filtro de género na prestação de serviços DSSR/VIH centrados em adolescentes e mulheres jovens.

Métodos: Uma revisão abrangente identificou estudos sobre intervenções e resultados em matéria de HIV em cenários de emergência, publicados em literatura científica reconhecida (2002-2017). Este exercício foi complementado com uma análise documental de orientação normativa, enquadramentos e diretrizes de implementação em matéria de VIH e SSR em intervenções de emergência, bem como consultas com especialistas no assunto.

Resultados: Os enquadramentos e orientações existentes prestam pouca atenção à saúde sexual e reprodutiva e aos direitos das mulheres jovens que vivem com VIH, centrando-se principalmente na prevenção da transmissão materno-infantil, na terapêutica antirretrovírica (TAR), nos serviços de despistagem do VIH e na articulação de serviços de tratamento. Aplicar um filtro de saúde sexual e reprodutiva de género à resposta proporciona oportunidades para identificar as questões de implementação importantes, bem como destacar práticas promissoras que se adaptam melhor aos serviços atuais para adolescentes e mulheres jovens.

Conclusões: As diversas necessidades concorrentes remetem para segundo plano o tempo e o espaço dedicados à integração eficaz das intervenções em matéria de VIH e saúde sexual e

reprodutiva em cenários de emergência. É necessário haver vontade política para fazer progredir a cooperação multissectorial, através de planeamento conjunto, formação sobre direitos humanos e respostas integradas, bem como promover soluções criativas para continuação de TAR, fornecimento de medicamentos e serviços de despistagem, tratamento e cuidados de saúde do VIH. Os últimos avanços em políticas e práticas sugerem que uma resposta centrada em adolescentes e mulheres jovens é viável.

Palavras-chave

VIH/SIDA, Adolescentes e mulheres jovens, Humanitária, Conflito, Crise, Desastre, Enquadramento, Emergência, TAR, Saúde sexual e reprodutiva, Diretrizes, Direitos

Sobre este suplemento

Este artigo foi publicado como parte da revista científica *Reproductive Health*, Volume 16, Suplemento 1, 2019: Integração Eficaz dos Serviços de Saúde Sexual e Reprodutiva e de Prevenção, Cuidados e Tratamento do VIH na África Subsariana: Onde estão as provas da implementação do programa?
O suplemento foi publicado como uma colaboração entre as revistas científicas *Reproductive Health* e *BMC Public Health.* O conteúdo integral, incluindo as versões em francês, português e inglês, estão disponíveis online:
https://bmcpublichealth.biomedcentral.com/articles/supplements/volume-19-supplement-1
e
https://reproductive-health-journal.biomedcentral.com/articles/supplements/volume-16-supplement-1
